# Production of red‐flowered oilseed rape via the ectopic expression of *Orychophragmus violaceus OvPAP2*

**DOI:** 10.1111/pbi.12777

**Published:** 2017-07-26

**Authors:** Wenqin Fu, Daozong Chen, Qi Pan, Fengfeng Li, Zhigang Zhao, Xianhong Ge, Zaiyun Li

**Affiliations:** ^1^ National Key Laboratory of Crop Genetic Improvement National Center of Oil Crop Improvement (Wuhan) College of Plant Science and Technology Huazhong Agricultural University Wuhan China; ^2^ Qinghai Academy of Agricultural and Forestry Sciences Qinghai University Xining China

**Keywords:** *Brassica napus*, flower colour, anthocyanin, transcriptome, *PAP2*, *Orychophragmus violaceus*

## Abstract

Oilseed rape (*Brassica napus* L.), which has yellow flowers, is both an important oil crop and a traditional tourism resource in China, whereas the *Orychophragmus violaceus*, which has purple flowers, likely possesses a candidate gene or genes to alter the flower colour of oilseed rape. A previously established *B. napus* line has a particular pair of *O. violaceus* chromosomes (M4) and exhibits slightly red petals. In this study, the transcriptomic analysis of M4, *B. napus* (H3), and *O. violaceus* with purple petals (OvP) and with white petals (OvW) revealed that most anthocyanin biosynthesis genes were up‐regulated in both M4 and OvP. Read assembly and sequence alignment identified a homolog of *AtPAP2* in M4, which produced the *O*.* violaceus* transcript (*OvPAP2*). The overexpression of *OvPAP2* via the CaMV35S promoter in *Arabidopsis thaliana* led to different levels of anthocyanin accumulation in most organs, including the petals. However, the *B. napus* overexpression plants showed anthocyanin accumulation primarily in the anthers, but not the petals. However, when *OvPAP2* was driven by the petal‐specific promoter XY355, the transgenic *B. napus* plants produced red anthers and red petals. The results of metabolomic experiments showed that specific anthocyanins accumulated to high levels in the red petals. This study illustrates the feasibility of producing red‐flowered oilseed rape, thereby enhancing its ornamental value, via the ectopic expression of the *OvPAP2* gene. Moreover, the practical application of this study for insect pest management in the crop is discussed.

## Introduction

Three major groups of pigments, betalains, carotenoids and anthocyanins, are responsible for the attractive natural display of flower colours (Grotewold, 2006). While carotenoids and betalains are generally yellow or red in colour, anthocyanins confer a diverse range of colours, from orange and red to violet and blue (Tanaka *et al*., 2010). There are six major classes of anthocyanidins, including pelargonidin, cyanidin, peonidin, delphinidin, petunidin and malvidin; although they constitute the core anthocyanidins that are predominant in nature, over 600 anthocyanins have been found, and these core anthocyanidins vary in their side chain decorations (Zhang *et al*., 2014a).

The anthocyanin biosynthetic pathways are well understood and are conserved among seed plants. Anthocyanins are formed from phenylalanine via phenylpropanoid metabolism, and their chemistry, biosynthesis and regulation have been described (Glover and Martin, 2012; Grotewold, 2006; Ramsay and Glover, 2005; Winkel‐Shirley, 2001). Anthocyanin production is controlled primarily at the transcriptional level. In the dicot *Arabidopsis*, the anthocyanin biosynthesis genes (ABGs) can be divided into early biosynthesis genes (EBGs), including *chalcone synthase* (*CHS*), *chalcone isomerase* (*CHI*), *flavanone 3‐hydroxylase* (*F3H*), *flavonoid 3′‐hydroxylase* (*F3′H*), *flavonol synthase* (*FLS*) and late biosynthesis genes (LBGs), including *dihydroflavonol 4‐reductase* (*DFR*), *anthocyanidin synthase* (*ANS*) and anthocyanidin 3‐*O*‐glucosyltransferase (*UFGT*). The EBGs are regulated by individual R2R3 MYB transcription factors (MYB11, MYB12, MYB111), whereas the LBGs are regulated by a MYB‐bHLH‐WD40 (MBW) complex, which is also found in many other dicot species, such as *Petunia hybrida*,* Zea mays* and *Antirrhinum majus* (Petroni and Tonelli, 2011). Several proteins belonging to the complex were shown to regulate anthocyanin biosynthesis in different species. For example in *Arabidopsis*, glabrous (GL3), enhancer of glabrous (EGL3) and transparent testa 8 (TT8) from the basic‐helix‐loop‐helix (bHLH) family; production of anthocyanin pigment 1 (PAP1), production of anthocyanin pigment 2 (PAP2), MYB113 and MYB114 from the MYB family; and transparent testa glabrous (TTG1), a WD40‐type protein, were found to play a role (Petroni and Tonelli, 2011; Ramsay and Glover, 2005).

*Brassica* crops (UN, 1935), particularly oilseed rape (OSR; *Brassica napus* L., 2n=38, AACC), cover many acres worldwide and are important sources of edible oil and biodiesel (Döring *et al*., 2012; Xia *et al*., 2016). In China, OSR is also considered an attractive ornamental crop, and several areas are well known for their OSR flower tourism, including Luoping County in Yunnan Province, Wuyuan County in Jiangxi Province, Jingmen County in Hubei Province, Menyuan County in Qinhai Province and Anshun County in Guizhou Province (Fu *et al*., 2016). OSR flower tourism has become an important part of the local economy. For example, in Wuyuan, which contains 1200 hectares of OSR, the income from tourism was approximately 2.896 billion CNY, accounting for 51.5% of the county's gross domestic product (GDP) in 2011 (Zhang, 2014). Massive amounts of multicoloured OSR flowers could further increase the aesthetic value of OSR as a tourism resource.

From another perspective, the large area used for OSR cultivation has far‐reaching implications for the severity of pest and disease problems and their consequent crop protection strategies (Döring *et al*., 2012). The pollen beetle *Meligethes aeneus*, which responds to coloured stimuli and prefers yellow to other colours, is a major pest of OSR at the inflorescence stage (Cook *et al*., 2013; Döring *et al*., 2012). Changing the colour of the petals would significantly reduce the attractiveness of the plant to *M. aeneus*; flowers that were dyed red were less heavily infested than those that were dyed blue; those dyed yellow and white flowers were most attractive (Cook *et al*., 2013). Therefore, the potential for the manipulation of petal colour as a new pollen beetle control strategy has been proposed, and the manipulation of anthocyanins for breeding of novel petal coloured OSR has been suggested (Cook *et al*., 2013).

However, to date, there are no reports on other flower colour variations (pink, red, purple, etc.) caused by the accumulation of anthocyanins in the petals, although red or purple colours are common in the leaves, stems, sepals and pods of these species (Mushtaq *et al*., 2016). The crucifer *Orychophragmus violaceus* (L.) O. E. Schulz (2n=24, OO genomes), which has large purple flowers, is widely cultivated as an ornamental plant in China. One disomic addition line with all the chromosomes of *B. napus* and a specific pair of *O. violaceus* chromosomes produced flowers with a red colour (Ding *et al*., 2014), suggesting that this foreign chromosome carried the key genes responsible for red petal colour. In this study, we used RNA‐Seq to perform comparative petal transcriptome profiling between the addition line, and the donors *B. napus* and *O. violaceus*, which have purple and white flowers. An *O. violaceus* homolog of *AtPAP2* (*OvPAP2*) in the anthocyanin biosynthetic pathway was identified as being expressed in the petals of the addition line. The ectopic expression of *OvPAP2* driven by a *Brassica* petal‐specific promoter, rather than its own promoter or the 35S promoter, successfully produced a red colour on the yellow petals of *B. napus*. These results could contribute to the production of OSR with entirely red flowers, optimizing OSR tourism resources and generating strategies for pest control. The potential regulation of anthocyanin accumulation in the petals of *Brassica* species is discussed.

## Results

### The phenotypes of four samples used for RNA‐Seq

**Figure 1:**
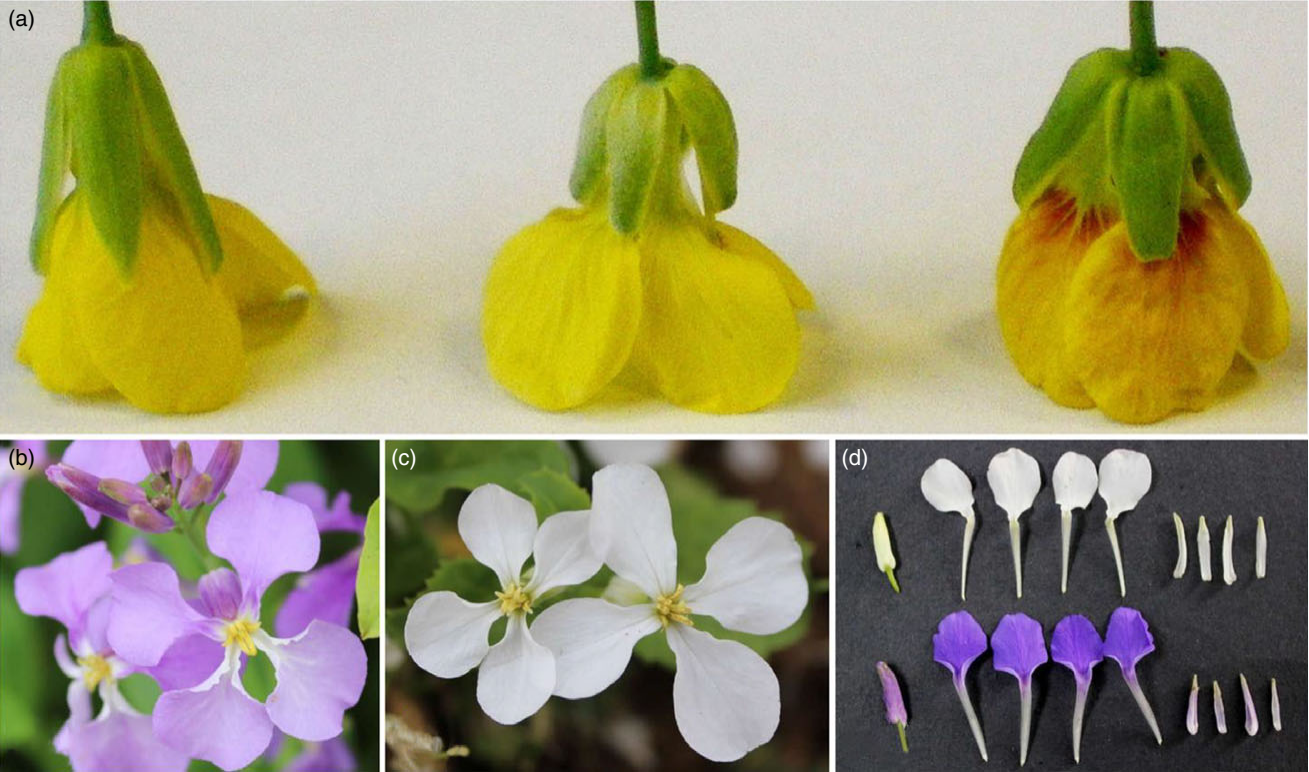
Floral phenotypes of *Brassica napus* and *Orychophragmus violaceus* of different colours. (a, left to right) Flowers of *Brassica napus*, monosomic and disomic addition lines. (b, c) Purple and white flowers of *Orychophragmus violaceus*. (d) White (top) and purple (bottom) flower buds and petals of *Orychophragmus violaceus*.

The disomic addition line (M4) plants with red petals had a phenotype that was nearly the same as that of the donor, *B. napus* cv. Huashuang 3 (H3), at the early vegetative growth stage, except for the purple colour on some of the youngest leaves and buds. At bolting, the stature of the M4 plants was smaller than that of H3, and the flowers displayed variable degrees of red colour on the yellow background of the petals. In M4 plants, red pigments were observed at the basal portion of petals on half‐open or open flowers that overlapped with the yellow background, which generated ‘orange‐red’ parts of the yellow petals (Figure 1a); by contrast, the H3 petals were yellow. Additionally, some M4 anthers (the anther walls but not the dehisced pollen) also showed different degrees of red coloration, but the sepals remained green. Interestingly, no red pigments were produced in the flowers of the monosomic addition line, which contained a single copy of the same chromosome (Figure 1a), suggesting that the chromosome dosage affected the synthesis of red pigments. The violet petals of wild‐type *O. violaceus* (OvP) slowly faded to lilac; in contrast, its natural mutant (OvW) had completely white petals (Figure 1b–d).

### The expression patterns of anthocyanin biosynthetic pathway genes were consistent with petal colour

To identify key genes responsible for the petal colour variation in M4, H3 and *O. violaceus*, we performed petal transcriptome analysis. In total, 14 G of raw data and 115 million high‐quality clean reads were obtained. For each of the four samples, approximately 30 million reads were obtained, 89% of which were clean reads (Table S1), highlighting the good quality of the library construction and sequencing. For M4 and H3, approximately 60% of the clean reads were mapped to the reference genome of *B. rapa,* but only 10% of the clean OvP and OvW reads were mapped (Table S1). A total of 37 441 expressed genes were identified from all four samples; 9917 exhibited different levels of expression between M4 and H3, and 3818 genes were differentially expressed between OvP and OvW. This result indicated that the difference in gene expression between M4 and H3 was much greater than that between OvP and OvW.

Previously, 73 genes in *B. rapa* were identified as orthologs of 41 ABGs in *A. thaliana* (Guo *et al*., 2014). Of these, 72 were identified in each sample in this study; the exception was *BrCPC2* (Bra039283) (Table S2). To simplify the comparison between different samples, for each gene in *Arabidopsis*, we added the expression values of all orthologous genes together. Most of the EBGs and LBGs were highly expressed in OvP and M4 (Figure S1), which were purple and red, respectively, compared with in OvW and H3, although the biosynthetic genes in the phenylpropanoid pathway were expressed at a low level (Figure S2). Notably, the key gene for anthocyanin synthesis, *ANS*, was completely silenced in H3 and showed almost no expression in OvW, which explained why there was no anthocyanin accumulation in the petals of H3 or OvW. Similarly, *DFR* and *TT19* were minimally expressed in H3 (Table S2).

In contrast to these structural genes, almost all regulatory genes were expressed at relatively low levels (Figure S3; Table S2); two exceptions were *PAP1* and *EGL3*, which showed higher expression in *O. violaceus* than in *B. napus*. Moreover, most genes showed higher expression in M4 than in H3 but almost the same expression in OvW and OvP (Figure S3). In detail, only one gene, *GL3* (Bra025508), showed significantly different expression in *O. violaceus*, with its fragments per kilobase of transcript sequence per million base pairs sequenced (FPKM) value being reduced from 15.13 in OvP to 0 in OvW (Table S2). In M4, except for *PAP1* (Bra039763), which had a slightly lower expression, seven other positive regulators (*MYB12* (Bra004456), *MYB111* (Bra037419), *PAP1* (Bra001917 and Bra004162), *TT8* (Bra037887), *GL3* (Bra025508), *EGL3* (Bra027653)) were up‐regulated. Moreover, two negative regulators (*MYBL2* (Bra016164), *LBD37* (Bra031833)) were down‐regulated, consistent with the colour change in the petals of M4 (Table S2). These results indicated that *PAP1* and *GL3* may be the key regulatory genes that contribute to the differential expression of LBG genes in the addition line and to the petal colour alteration in *O. violaceus*.

### Expression of *O. violaceus OvPAP2*, encoding an R2R3 MYB transcription factor, in M4

After the *de novo* assembly and comparison of all unigenes from *O. violaceus* and M4, 68 643 pairs of unigenes showed high similarity, but 104 pairs were annotated as ABGs (Table S3). However, only two unigene pairs (comp16133_c0_seq2 and comp26694_c0_seq4; comp16133_c0_seq1 and comp26694_c0_seq3) were annotated as *PAP2* genes showing high sequence similarity (>99%) and long BLAST lengths (>750 bp) between M4 and *O. violaceus*. This result indicated that these unigenes, which were expressed in M4, might be transcribed from the foreign chromosome from *O. violaceus*. Specific primers were designed to amplify the homologs of *PAP2* from M4 and *O. violaceus* (Table S4), and only one CDS each was identified in *O. violaceus* and in M4. An alignment of the CDSs with those from *Brassica rapa* (Bra039763), *Brassica oleracea* (Bol042409) and *A. thaliana* (AT1G66390) showed that the sequence from M4 was identical to that of *O. violaceus* (Figure S4). These results revealed that the added *O. violaceus* chromosome pair in M4 carried one *PAP2* homolog (*OvPAP2*), which was expressed in the petals, but the homologous *PAP2* genes from the *B. napus* genome were not expressed or were expressed at a very low level. The *OvPAP2* cDNA encoded a 238‐amino acid protein containing an R2R3 MYB domain with a signature motif for interaction with bHLH proteins from the 3f clade (DLX2RX3LX6LX3R; Figure S5a) (Butelli *et al*., 2012; Heim *et al*., 2003; Kui *et al*., 2010; Zimmermann *et al*., 2004). It also had a conserved KPXPR (S/T) F motif in its C‐terminal domain, which is found in other R2R3 MYB regulators of anthocyanin biosynthesis (Butelli *et al*., 2012; Kui *et al*., 2010; Stracke *et al*., 2001). Phylogenetic analysis revealed that *OvPAP2* clustered with *PAP1*,* PAP2*,* MYB113* and *MYB114*, which encode R2R3 MYB family members that are known to regulate anthocyanin biosynthesis in *Arabidopsis* (Gonzalez *et al*., 2008) (Figure S5b).

### CaMV35S‐driven overexpression of *OvPAP2* in *A. thaliana* and *B. napus*

**Figure 2:**
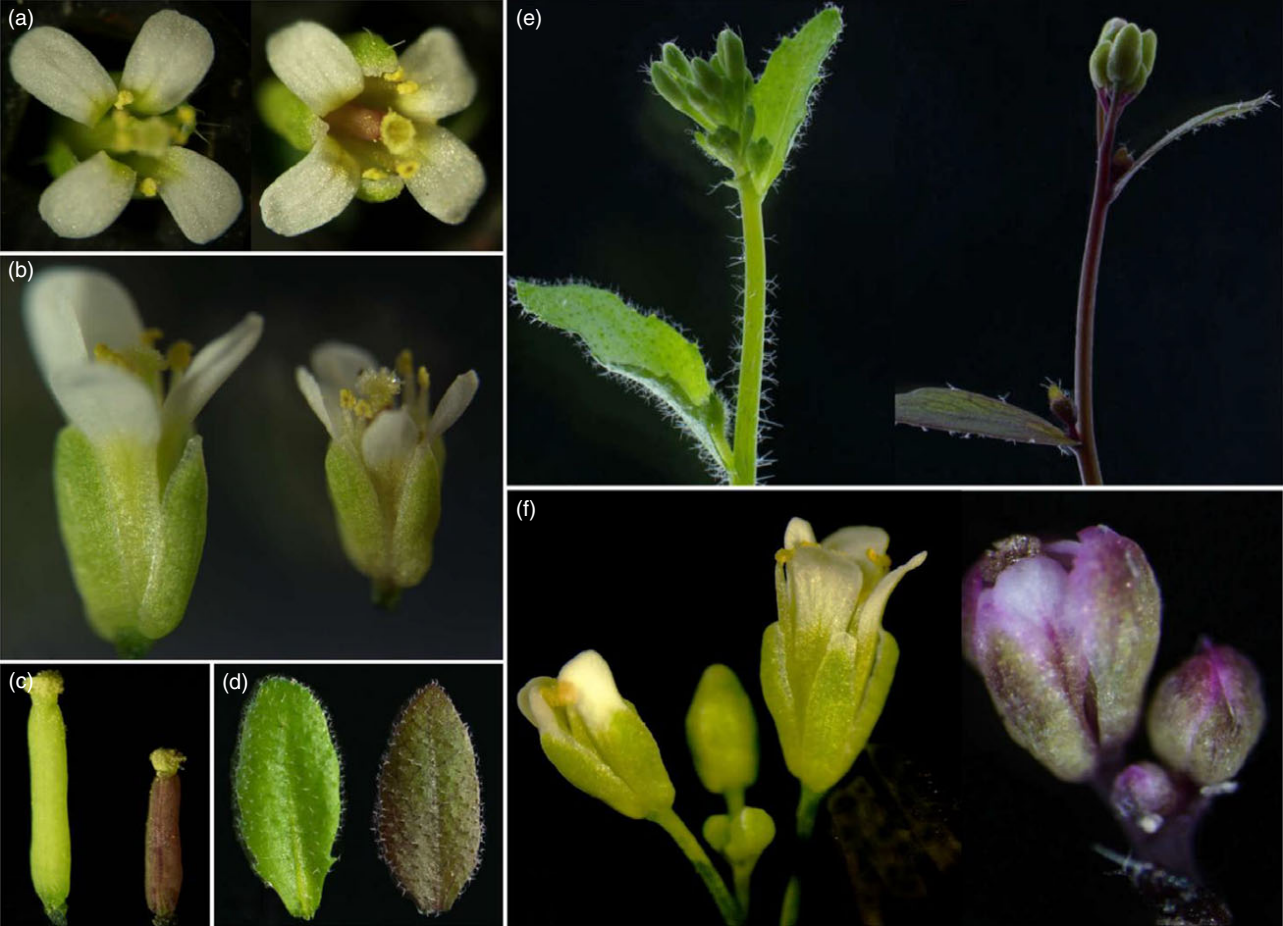
Phenotypes of *OvPAP2*‐overexpressing *Arabidopsis* plants. (a) and (b) Flowers. (c) Pistils. (d) Leaves. (e) Inflorescences. (f) When flowering declined, the flowers of the transgenic plants showed obvious purple petals and sepals. The wild‐type is on the left, and transgenic plants are on the right in all the panels.

An overexpression vector containing *OvPAP2* driven by the CaMV35S promoter was constructed, and after transforming wild‐type *A. thaliana* (Columbia), four transgenic plants with obvious purple phenotypes were obtained. The *OvPAP2*‐overexpressing plants showed varying levels of purple pigmentation on the stems and leaves, especially the pistils (Figure 2). In the flowers, in addition to the pistils and ovules, the anther walls and filaments showed a slight purple‐red colour, but the coloration on the petals and sepals was not obvious, with only a slightly purple strip on the base or in the middle (Figure 2a and b). However, as flowering declined, some smaller flowers showed a very obvious purple colour on the petals and sepals, especially on the margin of the sepals and in the middle of the petals (Figure 2f). These results indicated that the ectopic expression of *OvPAP2* in *Arabidopsis* promoted anthocyanin accumulation in different organs, including the petals.

**Figure 3:**
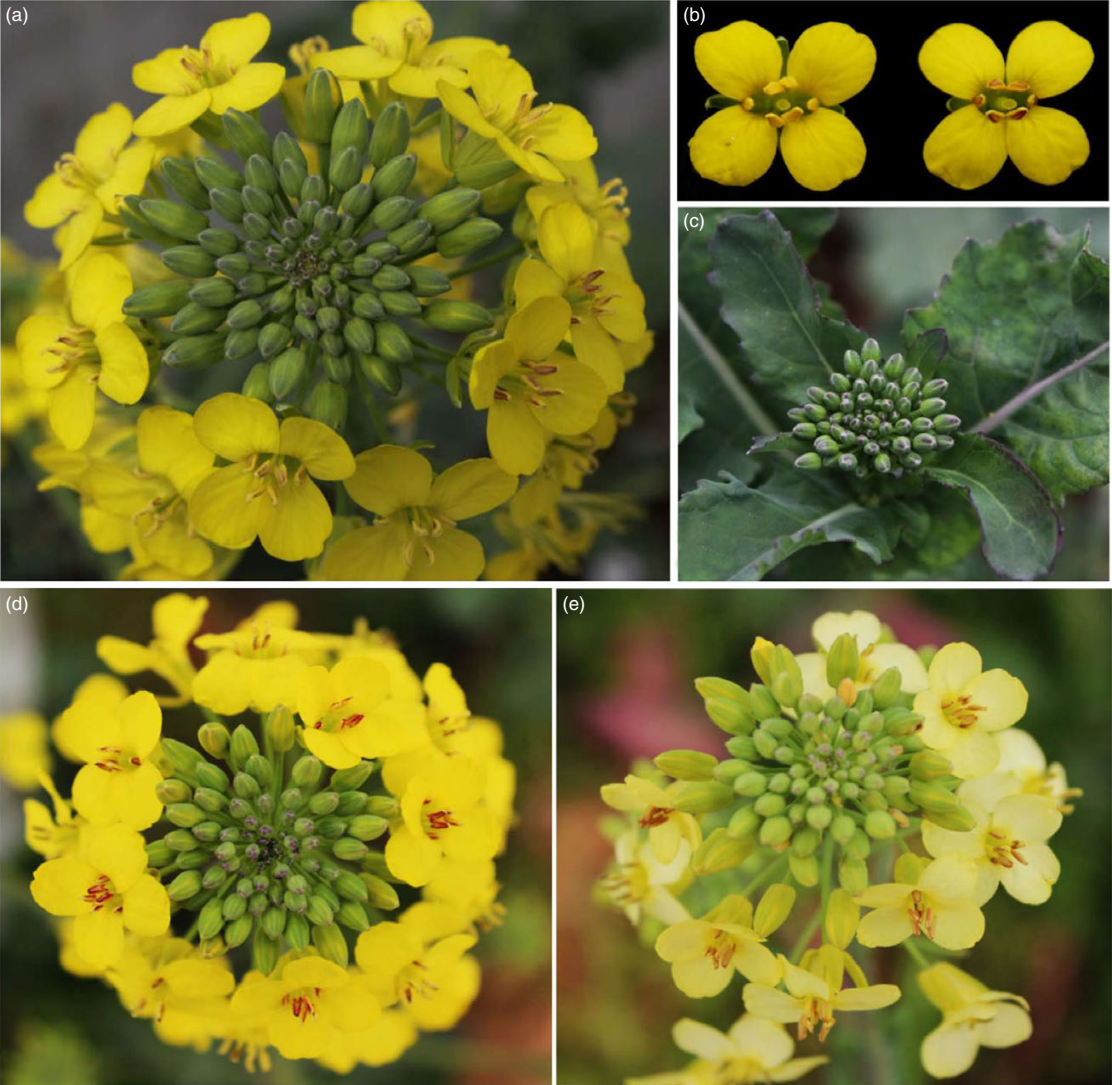
Phenotypes of *CaMV35S::OvPAP2 Brasica napus* plant and the offspring. (a) and (c) Inflorescence and young inflorescence of *CaMV35S::OvPAP2* T_0_ plant. (b) Flowers of Westar (left) and T_0_ plant (right). (d) Inflorescence of the T_1_ plant. (e) Inflorescence of an F_1_ plant of a cross between the *CaMV35S::OvPAP2* T_0_ plant and *Brassica napus* cv. G1300, which has white flowers.

**Figure 4:**
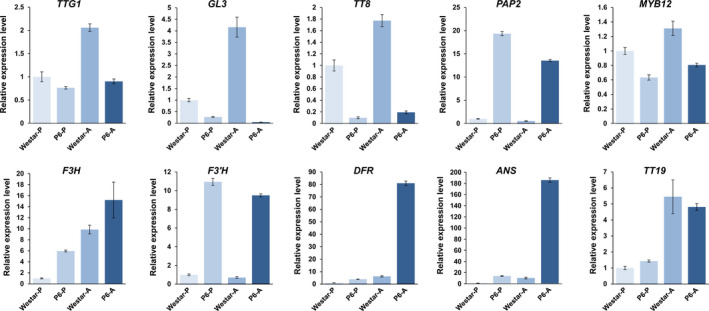
The relative expression levels of ten ABGs in the petals (P) and anthers (A) of *Brassica napus* cv. Westar and P6 based on qRT‐PCR. ABGs: anthocyanin biosynthesis genes; P6: *CaMV35S::OvPAP2* plant with red anthers.

We then transformed the vector into two *B. napus* varieties: H3 and *B. napus* cv. Westar, but only a few seedlings could be regenerated from Westar. Among the 29 *OvPAP2*‐overexpressing plants, ten displayed different degrees of red coloration on the anther walls (but not the pollen) and of purple on the youngest leaves and buds, but no purple or red colour was observed in the petals or other organs (Figure 3). The red anther walls were inherited in self‐crossed progenies and were much redder in some of the offspring plants (Figure 3d), but the petals were still pure yellow. To determine the reason why the *B. napus* petals failed to produce red pigments despite the overexpression of *OvPAP2*, we selected ten ABGs for the quantitative real‐time PCR (qRT‐PCR) analysis of petals and anthers from Westar and P6 (*CaMV35S*::*OvPAP2* plant with red anthers) (Figure 4). *PAP2*,* F3H* and *F3′H* were significantly up‐regulated in both the petals and anthers of the P6 plant compared with wild‐type *B. napus* cv. Westar. However, the key LBG genes *DFR* and *ANS* were significantly up‐regulated only in the anthers of P6, which was the reason for the lack of a red colour in the petals of the P6 plant. Interestingly, the other four regulatory genes were down‐regulated in P6 plants to varying degrees. To introduce the *OvPAP2* gene in a different genetic background, P6 was crossed with the white‐flowered *B. napus* cv. G1300 as the female parent. Again, the offspring had red anther walls but not red petals (Figure 3e). Furthermore, neither the petals nor the anthers of transgenic plants with the *OvPAP2* gene driven by the native promoter exhibited red coloration (data not shown).

### *OvPAP2* overexpression driven by a petal‐specific promoter produced red petals in *B. napus*

**Figure 5:**
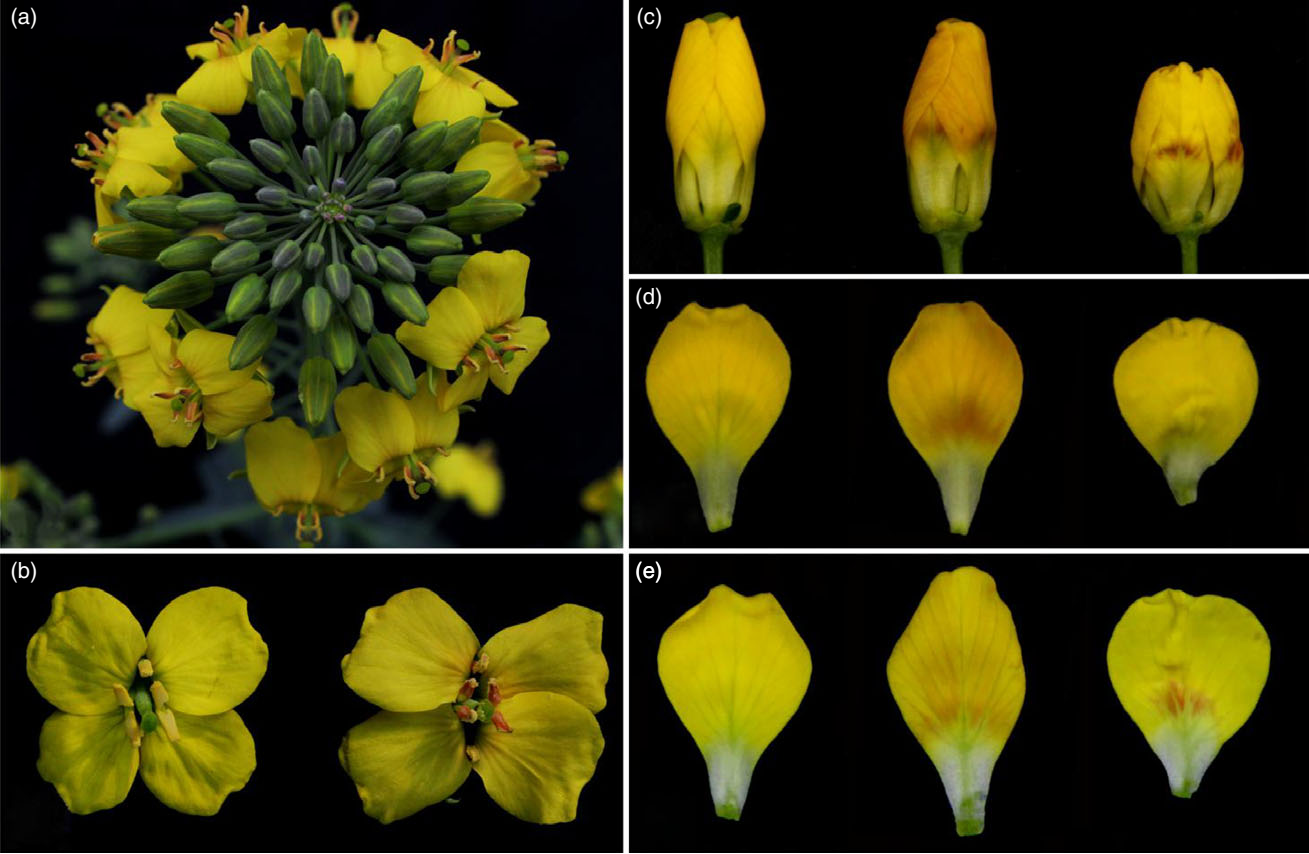
Phenotypes of the *XY355::OvPAP2 Brassica napus* plant. (a) Inflorescence. (b) Flowers of J9709 (left) and transgenic *Brassica napus* (right). (c) Flower buds after removing the sepals. (d) and (e) Adaxial and abaxial sides, respectively, of petals on the flower bud in c. (c–e) From left to right: J9709, transgenic *Brassica napus* and M4.

**Figure 6:**
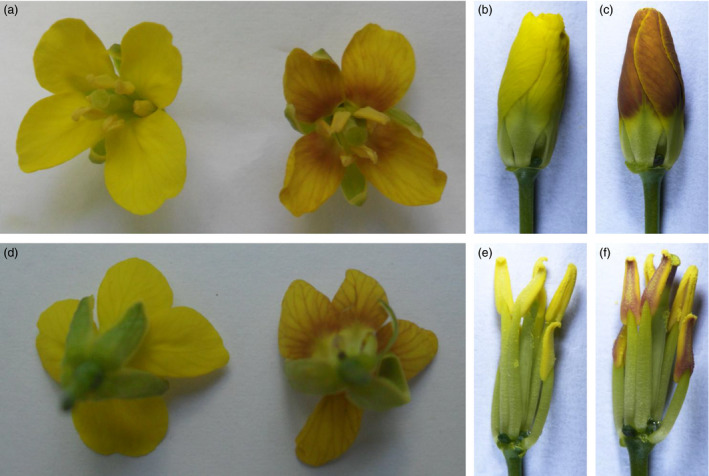
Phenotypes of individual T_1_ *XY355::OvPAP2 Brassica napus* plants. (a) and (d) Adaxial and abaxial sides of the flowers, respectively. (b) and (c) Flower buds after removing the sepals. (e) and (f) Flower buds after removing the sepals and petals. (a) (left), (b), (d) (left) and (e) T_1_ plant without red petals and anthers. (a) (right), (c), (d) (right) and (f) T_1_ plant with red petals and anthers.

**Figure 7:**
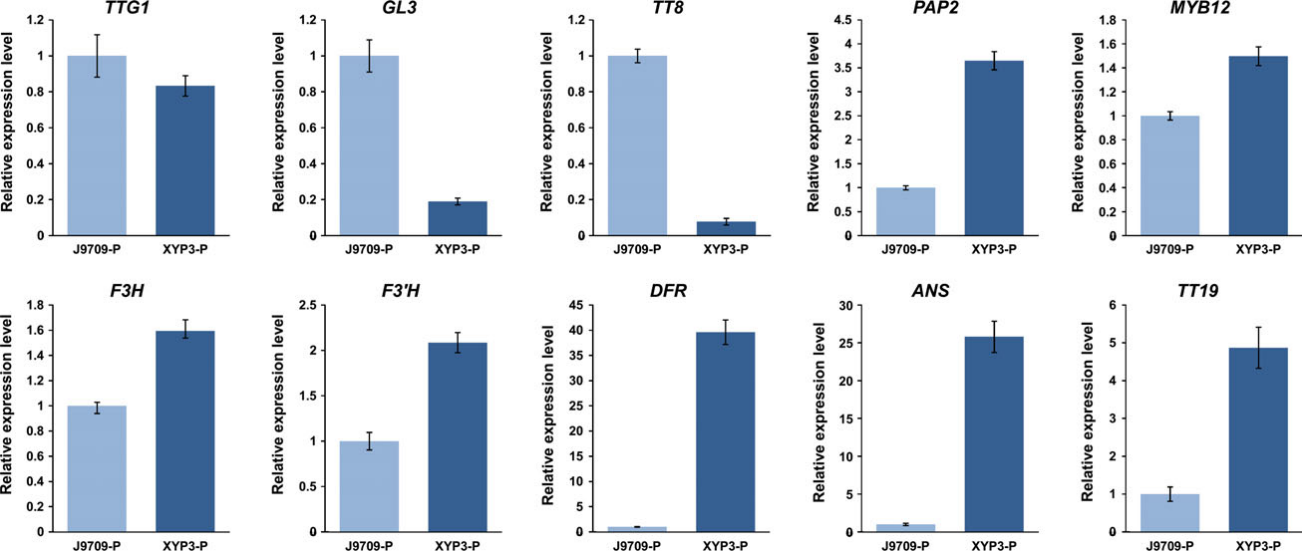
The relative expression levels of ten ABGs in the petals (P) of *Brassica napus* (J9709 and XYP3) based on qRT‐PCR. ABGs: anthocyanin biosynthesis genes; XYP3: *XY355::OvPAP2* plant.

**Figure 8:**
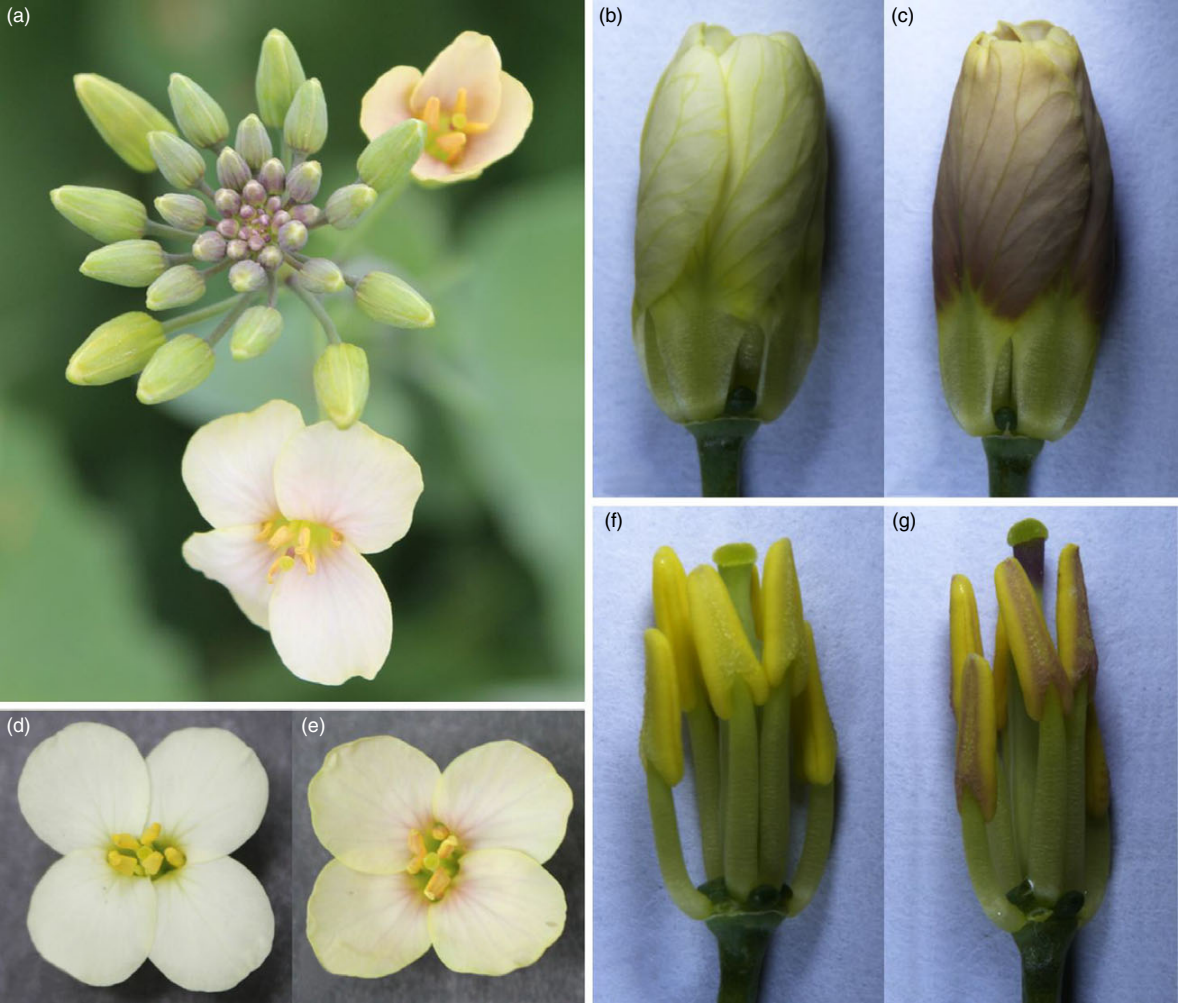
Phenotypes of F_1_ individuals from a cross between a *XY355::OvPAP2 Brassica napus* plant and *Brassica napus* cv. G1300, which has white flowers. (a) Inflorescence. (b) and (c) Flower buds after removing the sepals. (d) and (e) Flowers. (f) and (g) Flower buds after removing the sepals and petals. (a), (c), (e) and (g) F_1_ plant with red petals and anthers. (b), (d) and (f) F_1_ plant without red petals and anthers.

The petal‐specific promoter XY355 was amplified from the genome of *B. napus* (H3) according to sequence information (Brocard *et al*., 2001). An overexpression vector containing *OvPAP2* driven by the XY355 promoter was constructed and transformed into another *B. napus* variety, J9709. Of the 31 total regenerated plants, two were positive transgenics and produced red pigments in both the anther walls (but not the pollen) and the petals (Figure 5) while retaining the other morphological characters of the recipient J9709. The red phenotype of the *XY355*::*OvPAP2* (XYP3) petals was more stable and consistent among different flowers than was that of the M4 petals. Similarly, the red pigments mainly accumulated at the base of the petals and were most obvious in the semi‐open or open flowers (Figure 5c). However, the distribution of pigments on the petals differed slightly between M4 and the transgenic plants, as the red pigments were found on both the adaxial and abaxial sides of the *XY355*::*OvPAP2* petals but only on the abaxial side of the M4 petals (Figure 5d and e). The other parts of the *XY355*::*OvPAP2* petals also accumulated a small amount of red pigment, which made the entire flower appear orange (Figure 5c). After self‐pollination, the offspring (T_1_) of the *XY355*::*OvPAP2* plants produced much redder flowers, which generated more red pigments at the base of the petals as well as in other parts, especially the vascular tissue, resulting in petals with a red texture (Figure 6). The T_2_ individuals showed a still stronger phenotype and even showed a small amount of purple on the sepals (Figure S6a, c and d). qRT‐PCR analysis clearly indicated that *PAP2*,* MYB12*,* DFR*,* ANS*,* F3H*,* F3′H* and *TT19* were significantly up‐regulated in the petals of *XY355*::*OvPAP2* plants. Again, three regulatory genes, *GL3*,* TT8* and *TTG1*, were down‐regulated to varying degrees, as in the P6 plants (Figure 7). The red colour of the anthers meant that this promoter was active. The *XY355*::*OvPAP2* T_0_ plant was also crossed with the white‐flowered cultivar *B. napus* cv. G1300 as the male parent. The resulting offspring (F_1_) produced slightly red/purple pigments both on the anther walls and at the base of the petals (Figure 8). Similarly, the F_2_ individuals showed more obviously red/purple flowers (Figure S6b, e and f).

### The expression of *PAP2* was closely associated with anthocyanin accumulation in the anthers and petals of *B. napus*

**Figure 9:**
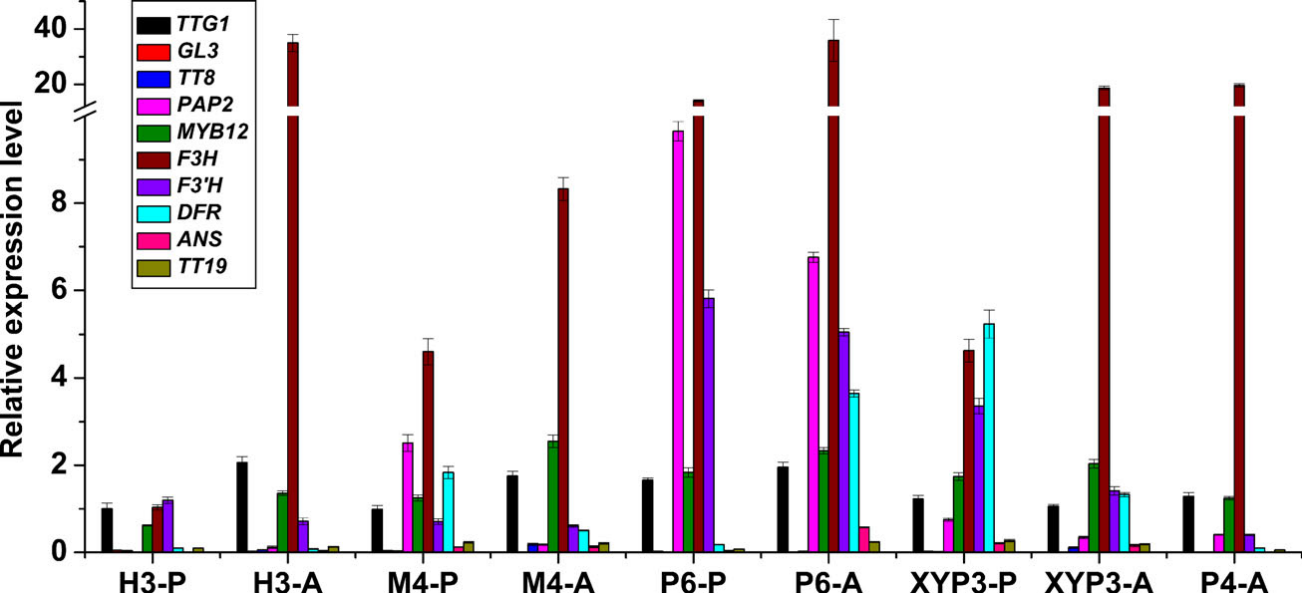
The relative expression levels of ten ABGs in the petals (P) or anthers (A) of different *Brassica napus* samples. P6: *CaMV35S::OvPAP2* plant with red anthers; XYP3: *XY355::OvPAP2* plant; P4: *CaMV35S::OvPAP2* plant without a phenotype; ABGs: anthocyanin biosynthesis genes.

To further observe and study the reason for the different phenotypes of the different materials, the ten ABGs were analysed by qRT‐PCR in the petals and/or anthers of H3, M4 and P4 (*CaMV35S*::*OvPAP2* plant without a phenotype), in addition to P6 and XYP3 (Figure 9). The following results were obtained: (i) most of the genes showed the same expression trends in the petals of M4 and H3, as revealed by qRT‐PCR and RNA‐Seq, suggesting the reliability of the RNA‐Seq data; (ii) *PAP2* was significantly up‐regulated in both the petals and anthers of the P6 and M4 plants when compared with H3; however, the key LBG genes *DFR* and *ANS* were significantly up‐regulated only in the anthers of P6, and *ANS* showed almost no expression in the petals, which explained the lack of red pigments in the petals; (iii) *PAP2* expression was up‐regulated in *XY355*::*OvPAP2* petals and anthers but was much lower than in M4 (petals) and P6 (petals and anthers); nevertheless, both *DFR* and *ANS* were up‐regulated in the two tissue types and were expressed more highly in *XY355*::*OvPAP2* petals and anthers than in M4 (petals and anthers) and P6 (petals), corresponding to the depth of the red colour in these materials; and (iv) *PAP2* expression increased slightly in the anthers of the P4 plant, but the other genes were more or less the same in the anthers of H3, suggesting that the level of ectopic *OvPAP2* expression was important for regulating the structural genes in anthers. Based on these results, *OvPAP2* was considered important for anthocyanin biosynthesis in the red petals of M4, as it could up‐regulate several important structural genes in some tissues. However, only when both *DFR* and *ANS* were up‐regulated could the tissues produce red pigments, becoming redder with higher expression. Moreover, in the petals of transgenic plants, high *OvPAP2* expression under the petal‐specific promoter XY355 but not the CaMV35S promoter could up‐regulate both *DFR* and *ANS*, but the reason why is unclear.

### The composition of anthocyanins contributing to the red colour in petals

**Figure 10:**
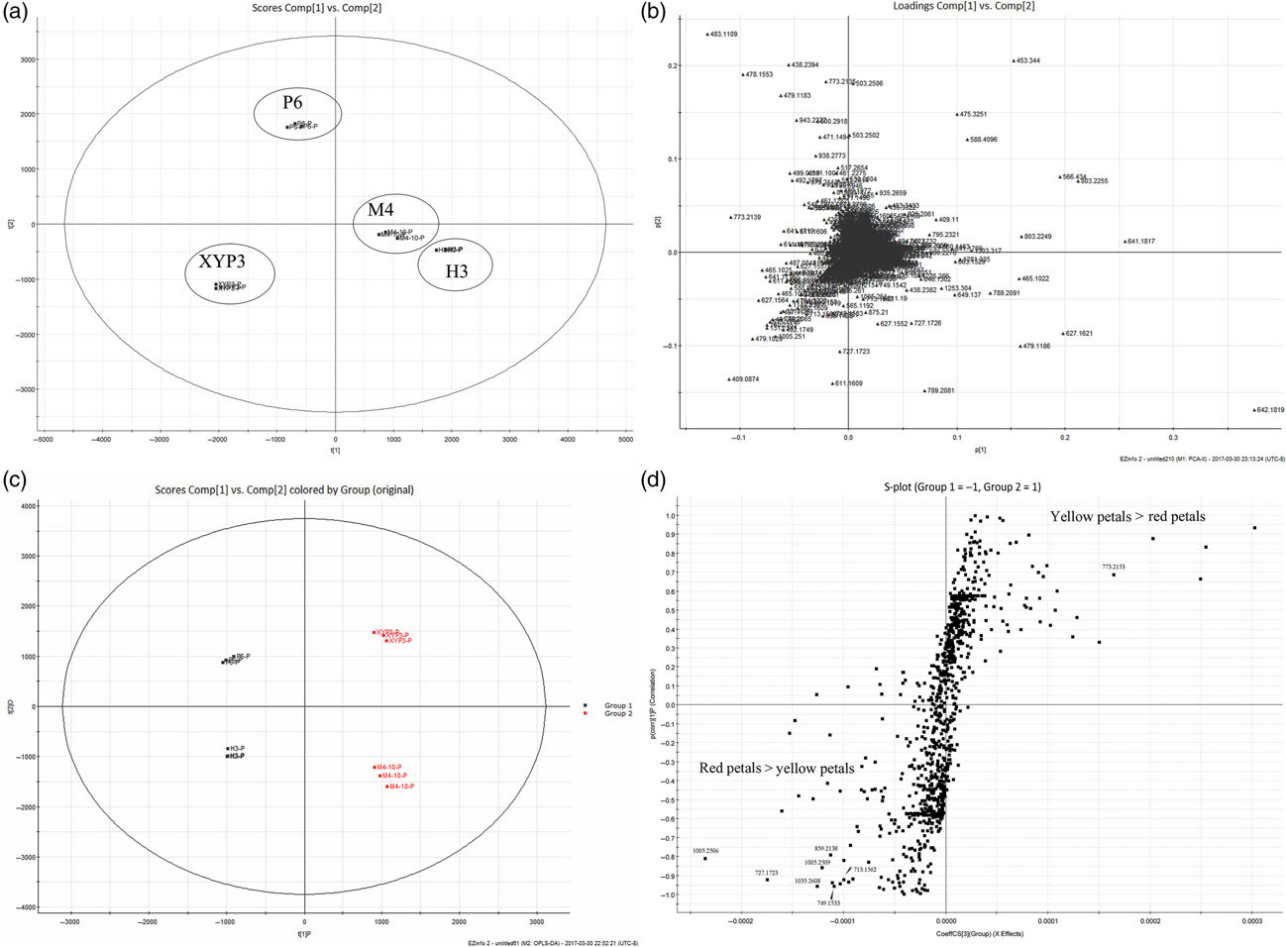
Principal component analysis (PCA) and orthogonal projection to latent structure‐discriminant analysis (OPLS‐DA) of petals of *Brassica napus* (M4, XYP3, H3 and P6). (a, b) Unsupervised PCA score plots and loading plot of four samples. (c, d) OPLS‐DA score plots and loading S‐plots of red petals (M4 and XYP3) vs. yellow petals (H3 and P6). Potential biomarkers for grouping were identified by analysing the S‐plot, which was plotted using covariance (p) and correlation (pcorr). Biomarkers indicated by molecular weight represent compounds with the most positive or negative VIP values of identified anthocyanins. XYP3: *XY355::OvPAP2* plant; P6: *CaMV35S::OvPAP2* plant with red anthers; VIP: variable importance in the projection.

To identify the main metabolites contributing to the red colour of the petals, we performed metabolite analysis on petals from H3, M4, P6 and XYP3. Principal component analysis (PCA) of the profiling data sets separated the four samples from each other (Figure 10a). The metabolites of P6 and XYP3 were markedly different from each other and from M4 and H3, while H3 and M4 were more similar, consistent with M4 and H3 having similar genetic backgrounds. The corresponding loading plot (Figure 10b) shows features (m/z ions) contributing clustering seen on the PCA plots.

To identify the metabolites that significantly differentiated the red petals (M4 and XYP3) from the yellow petals (H3 and P6), orthogonal projection to latent structure‐discriminant analysis (OPLS‐DA) modelling was performed on the profiling data sets (Figure 10c). S‐plots were then constructed by plotting covariance (p) against correlation (pcorr) (Figure 10d). The potential biomarkers for the separation of the yellow and red colours were obtained by filtering by variables important in the projection (VIP) > 2. Forty potential biomarkers were identified, 21 of which were abundant in red petals, while 19 were abundant in yellow petals. To identify the potential anthocyanins that contributed to the red colour, the tandem mass spectrometry (MS/MS) fragmentation patterns (m/z) of each biomarker were used for comparison with those radical groups found in previous studies (Wu and Prior, 2005). In total, 12 anthocyanins were identified, including cyanidin and delphinidin (Table S5). Surprisingly, the yellow petals also contained two types of anthocyanins (Table S5). The most preferentially accumulated anthocyanin in red petals showed peaks at retention times of 9.9 and 10.4 and a molecular weight of 1005.2124 (Figure S7a). MS data ([M]+, *m/z* 1005; MS/MS, *m/z* 757/535/287) indicated that it was a known anthocyanin, cyanidin 3‐coumaroyldiglucoside‐5‐malonylglucoside (Figure S7b) (He *et al*., 2016; Sun *et al*., 2013; Zhang *et al*., 2014b). One new anthocyanin with a molecular weight of 713.1562 was found to be delphinidin‐3‐malonyl glucoside‐5‐glucoside, according to the MS data ([M]+, *m/z* 713; MS/MS, *m/z* 551/465/303), but this identification requires further confirmation by other methods. These results indicated that red petals contained more anthocyanins than yellow petals and that some anthocyanins were red petal‐specific, which is in accordance with the higher expression of *F3H*,* F3′H*,* DFR* and *ANS*, which are involved in the key steps of anthocyanin production, in the petals of M4 and XYP3.

## Discussion

### Genetic regulation of petal colour in *O. violaceus* and *B. napus*

In the white petals of OvW plants and the red‐yellow petals of M4 plants, most of the EBG and LBG genes, as well as the transport gene *TT19*, were significantly down‐regulated when compared with the purple‐flowered OvP and up‐regulated when compared with the yellow‐flowered H3. These results suggest that the inactivation or activation of a transcription factor, rather than a single structural gene, may contribute to the down‐ or up‐regulation, respectively, of multiple genes in the anthocyanin pathway in OvW and M4, respectively. Correspondingly, the only differentially expressed regulatory gene, *GL3*, was down‐regulated to 0 in OvW. In *Arabidopsis*,* GL3* is a member of *bHLH* gene family and encodes one of the components of the MBW complex. Many studies have demonstrated that *GL3* is necessary for anthocyanin accumulation (Feyissa *et al*., 2009; Nukumizu *et al*., 2013). Thus, the only nonexpressed regulatory gene, *GL3,* was likely to be responsible for the down‐regulation of structural genes and anthocyanin deficiency in the petals of OvW plants, but further studies should be conducted to confirm this hypothesis. Similarly, differentially expressed gene (DEG) analysis between M4 and H3 and subsequent gene cloning and sequencing comparisons identified a homolog of *AtPAP2* from *O. violaceus* in M4. *AtPAP2* is a member of the *R2R3 MYB* gene family and is also a component of the MBW complex. The overexpression of either *PAP1* or *PAP2* could cause different degrees of anthocyanin production in the leaves, stems and flowers of *Arabidopsis* (Borevitz *et al*., 2000). The overexpression of *OvPAP2* via the OSR petal‐specific promoter XY355 could indeed generate both red petals and anthers in *B. napus*. Consequently, we hypothesized that the more highly expressed *OvPAP2* might be the key factor up‐regulating most EBGs and LBGs, resulting in red petals in M4. In other words, the inactivation of the tissue‐specific expression of *PAP2* during its evolutionary history might be the reason for the loss of pigmentation in *B. napus* petals.

Interestingly, the OvW plant used in this study is a spontaneous mutant. Mutations affecting transcriptional regulatory proteins are preferentially fixed in the evolutionary loss of pigmentation. In particular, natural selection left mutants with almost all floral R2R3 MYB transcription factors inactivated, eliminating floral anthocyanins, while spontaneous mutants with white flowers are often caused by inactivated bHLH and WDR proteins (Sobel and Streisfeld, 2013; Wessinger and Rausher, 2012). This pattern might occur because floral pigments are typically regulated by R2R3 MYB transcription factors that have tissue‐specific expression profiles (Albert *et al*., 2011; Ramsay and Glover, 2005); thus, the inactivation of the R2R3 MYB eliminated the anthocyanins in that tissue only. By contrast, the enzymes of the anthocyanin pathway as well as its bHLH and WDR regulators typically have broad expression domains and are responsible for the biosynthesis of anthocyanins and other flavonoids. Amino acid mutations in the pathway enzymes and the inactivation of these transcription factors will give rise to extensive deleterious pleiotropic mutations (Sobel and Streisfeld, 2013; Wessinger and Rausher, 2012). Correspondingly, we have observed that OvW plants have lost their pigments not only in the floral organs but also in the leaves and stems and were weaker than OvP plants (Chen DZ, Pan Q, Ge XH and Li ZY unpublished data).

### Delphinidin‐based anthocyanin production in *Brassica* species

In the anthocyanin biosynthesis pathway, the number of hydroxyl groups on the B‐ring plays a key role in determining pigment colour; when there are more ‐OH rings, the pigment is bluer (Tanaka and Brugliera, 2013). The hydroxylation pattern is determined by two cytochrome P450s, flavonoid 3′‐hydroxylase (F3′H) (classified as CYP75B) and flavonoid 3′, 5′‐hydroxylase (F3′5′H) (mainly CYP75A); these proteins lead to the generation of cyanidin‐based anthocyanins, which contribute to red and pink flower colour and to delphinidin‐based anthocyanins, which tend to have violet and blue colours (Tanaka and Brugliera, 2013). In *Arabidopsis*, the sequences of the *CYP75A* subfamily are not represented in the genome, which explains the absence of delphinidin‐based anthocyanins in *Arabidopsis* (Bak *et al*., 2011). The same is true for *B. rapa*,* B. oleracea* and *B. napus*, whose genomes do not contain *CYP75A* sequences (Chen *et al*., unpublished data). However, delphinidin‐based anthocyanins were previously found in *B. rapa* (purple bok choy) (Zhang *et al*., 2014b) and purple heading Chinese cabbage (He *et al*., 2016) as well as in the present study in *B. napus*, in which three types of known delphinidin and one type of new delphinidin were identified in red petals and one type of delphinidin was identified in yellow petals. Thus, it was interesting to investigate whether *B. rapa* and *B. napus* contain isoenzymes that share the same catalytic activity as F3′5′H. For example, some Asteraceae species have re‐acquired F3′5′H function from their *F3′H* gene by convergent evolution (Seitz *et al*., 2006).

### Flower colour modification for ornamental value and pest control

In the past 20 years, considerable progress has been made in the production of desirable and novel flower colours via the genetic engineering of important floricultural plants (Nishihara and Nakatsuka, 2011; Noda *et al*., 2013; Tanaka *et al*., 2010; Wessinger and Rausher, 2012). As an important economic crop, OSR is cultivated in large areas, especially in China; additionally, its ornamental value due to its golden yellow flowers is emerging as evidenced by sightseers visiting several areas of China where it is grown en masse. The alteration of petal colour was largely attributable to the R2R3 MYB transcription factor OvPAP2, and *OvPAP2* overexpression by genetic engineering resulted in redder petals in *B. napus* plants, increasing the visual appeal of OSR. Other coloured petals, such as purple and blue petals, that require the anthocyanin biosynthesis pathways could be obtained via the same technique by controlling the relative expression levels of key genes. To eliminate public concern about transgenic plants, a transformation method for obtaining marker‐free plants could be used (de Vetten *et al*., 2003).

OSR is known to be self‐fertile, but insects can spread its pollen and increase its seed set (Mänd *et al*., 2010); therefore, the effect of red‐flowered OSR on pollinators should be considered. However, the attractiveness of the red‐flowered OSR to bees would be unaffected because the red flowers are not completely invisible to bees (Chittka and Waser, 1997), and the signals from pollen, nectaries and floral volatiles are still present, which should be attractive to bees and facilitate pollination (Cook *et al*., 2005; Menzel, 1985).

The alteration of petal colour has significant effects on plant colonization by *M. aeneus*, and red flowers are less heavily infested than yellow flowers; thus, the manipulation of petal colour could have potential in new pest control strategies (Cook *et al*., 2013). The novel red‐ or blue‐flowered OSR could confer increased and long‐lasting resistance to *M. aeneus*, thus reducing reliance on insecticides and furthering the potential to incorporate these plants into environmentally benign control strategies (Cook *et al*., 2013).

## Conclusion

Here, a homolog of *AtPAP2* (*OvPAP2*), which encodes a key regulator in the MBW complex, was isolated from an *O. violaceus* chromosome added to *B. napus* by comparative transcriptomic analysis. The overexpression of *OvPAP2* via the 35S promoter in *Arabidospis* led to pigment accumulation in various organs, but in *B. napus*, it produced only red anthers. The petal‐specific promoter‐mediated overexpression of *OvPAP2* in *B. napus* successfully changed the yellow and white petals into red‐yellow/red petals due to the accumulation of known and new anthocyanins. The present study illustrated the feasibility of diversifying the flower colours of *B. napus* by introducing key gene(s) from other species.

## Experimental procedures

### Plant materials and RNA preparation

A disomic addition line with all the chromosomes of *B. napus* and one specific pair of *O. violaceus* chromosomes producing slightly red petals (designated M4) was obtained previously (Ding *et al*., 2014; Zhao *et al*., 2008). The M4 plant, the donor *B. napus* cv. Huashuang 3 (H3), the wild‐type *O. violaceus* with purple petals (OvP) and its natural mutant with white petals (OvW) were grown in the greenhouse of the Huazhong Agricultural University in the autumn of 2011. The petals of half‐open flowers were collected, and total RNA was extracted using the guanidine isothiocyanate method. The quality and quantity of the purified RNA were determined by measuring the 260 nm/280 nm absorbance (A260/A280) using a spectrophotometer (SmartSpec plus, Bio‐Rad, Hercules, CA). RNA integrity was further verified by 1.5% agarose gel electrophoresis. The total RNA of all samples was assessed based on A260/A280 ≈ 1.7, quantity > 10 μg and good integrity.

### RNA‐Seq library construction, sequencing, read mapping and DEG screening

For each sample, 10 μg of total RNA was used for RNA‐Seq library preparation. The libraries were prepared following the manufacturer's instructions and run on an Illumina HiSeq 2000 system for 100‐nt pair‐end sequencing by Majorbio. Inc. (Shanghai, China). After sequencing, the high‐quality clean reads were obtained by filtering the dirty reads with adaptors or two Ns and the low‐quality reads. The clean reads from each sample were mapped to the genome of *B. rapa* (http://brassicadb.org/brad/downloadOverview.php) using Tophat (Trapnell *et al*., 2012), allowing no more than two mismatched bases. Mapped reads were normalized using the DESeq Bioconductor package (Anders and Huber, 2010). Gene expression levels were calculated in terms of FPKM using Cufflinks and Cuffdiff (Trapnell *et al*., 2012; Zhu *et al*., 2010). DEGs between samples were determined using the edgeR program (Robinson *et al*., 2010) using the standards of *P*‐value ≤0.01 and fold change ≥2 or ≤0.5.

### *De novo* assembly of petal transcriptomes and sequence alignment

*De novo* assembly and comparative transcriptomic analysis were performed: all clean reads generated from two *O. violaceus* samples were assembled together as transcripts of *O. violaceus* using Trinity with default parameters (Haas *et al*., 2013), while clean reads of M4 were assembled alone. The unigenes were first annotated based on the TAIR database (http://www.arabidopsis.org/). The same unigenes expressed in M4 and *O. violaceus* were identified by performing an all against all DNA sequence similarity (BLASTN with an E‐value cut‐off of 1E‐5) BLAST.

### Cloning of *PAP2* and phylogenetic analysis

Based on the assembled sequence and the genome sequence of *B. napus* and *A. thaliana*, respectively, specific primers (Table S4) were designed to amplify the *PAP2* homologs from M4 and *O. violaceus* using Primer 3.0. Specifically, PAP2‐1F and PAP2‐1R were used to amplify *PAP2* from M4 petal cDNA and *O. violaceus* leaf DNA, respectively. Ex Taq (Takara Bio Inc., Otsu, Japan) was used for all fragment amplifications. The PCR products were cloned into pMD™18‐T (Takara, Dalian, China) and sequenced. The obtained sequences were BLASTed using SeqMan to identify the correct CDS of *PAP2*, which was mapped from different materials and species using ClustalW2 (http://www.ebi.ac.uk/Tools/msa/clustalw2/). The sequence alignment figures were made using GeneDoc.

The CDSs of MYBs belonging to subgroups 2, 4, 5, 6 and 7 from *Arabidopsis* were used to calculate distance estimates for a neighbour‐joining tree with MEGA6 software (Tamura *et al*., 2013). To provide statistical support for each node in the tree, a consensus tree was generated from 1000 bootstrap data sets. The protein sequences of the selected MYB proteins from *A. thaliana* and other species belonging to subgroup 6 were aligned online (http://multalin.toulouse.inra.fr/multalin/) to identify bHLH protein binding sites and conserved motifs defining the members of R2R3 MYB subgroup 6.

### Plasmid construction and genetic transformation

Plasmid vector construction for overexpression via genetic transformation was performed as described (Xia *et al*., 2016). Based on determinate sequence information, *OvPAP2* (was also the expressed *PAP2* in M4) was cloned from the cDNA of M4 into the pMDC83 containing the CaMV35S promoter using the traditional digestion and ligation method with a restriction endonuclease and T4 ligase (Thermo Scientific, (EU) Lithuania). The amplification primers were PAP2‐XF and PAP2‐BR (Table S4). The plasmid vector with the correct target sequence was transformed into *Agrobacterium* GV3101 for the genetic transformation of *A. thaliana* (Columbia) and *B. napus* (H3 and Westar). Another vector (pCAMBIA2300) containing *OvPAP2* was constructed (primers: PAP2‐PF and PAP2‐KR) with the OSR petal‐specific promoter XY355 (355 bp), which was amplified (primers: XY355‐HF and XY355‐PR) from the genome of *B. napus* (H3) according to sequence information (Brocard *et al*., 2001) and transformed into another *B. napus* variety, J9709, using the same method. The consensus primers for positive clone detection in this study were RV‐M, M13‐47 and pMDC83‐nos (Table S4). *Agrobacterium‐*mediated genetic transformation was used for both *A. thaliana* and *B. napus*. *A. thaliana* was transformed using the floral dip method (Clough and Bent, 1998), and *B. napus* was transformed using the hypocotyl infection method as previously described (Xia *et al*., 2016).

### qRT‐PCR analysis

Total RNA from several tissues, including the petals and anthers of different plant samples, was extracted for qRT‐PCR analysis using TRIzol reagent (Invitrogen, Carlsbad, CA) according to the user manual. First‐strand cDNA synthesis was performed with two micrograms of total RNA from each sample using a RevertAid™ First Strand cDNA Synthesis Kit (Thermo Scientific, (EU) Lithuania) according to the manufacturer's protocol. The specific quantitative primers for ten ABGs were designed using Primer 3.0; all primer sequences are listed in Table S6. qRT‐PCR assays with three replicates were performed using a KAPA SYBR FAST qPCR kit (Kapa Biosystems, Boston, MA) on a Bio‐Rad CFX96 Real‐Time Detection System. The *Bnaactin3* gene was used as an internal control for data normalization, and quantitative variation in the different replicates was calculated using the delta‐delta threshold cycle relative quantification method as described previously (Fu *et al*., 2014).

### Sample preparation and metabolomics analysis

The petals of newly fully opened flowers from H3, M4 and the overexpression plants were collected in liquid nitrogen. A total of 0.1 g of frozen powder was placed into a 2‐mL screw‐cap tube with 1 mL of methanol/water/acetic acid (85:15:0.5; v/v, MeOH/H2O/AcAc). The sample was then put on ice in the dark with sonication for 30 min and then vortexed at 4 °C in the dark overnight. The supernatant was collected after centrifuging at 12 000 × g for 10 min and was further diluted appropriately and filtered twice using a 0.22 μm PTFE filter (Cameo 25F, Micron Separations Inc., Westboro, MA) before injection for metabolomics analysis.

Metabolomics analysis was performed on a Waters Acquity UPLC system connected to a Synapt G2‐XS QToF mass spectrometer, which was equipped with an electrospray ionization source (Waters, Milford, MA), according to methods previously described (Jing *et al*., 2017). The UPLC was fitted with an Acquity UPLC BEH C18 column, 1.7 μm, 2.1 × 150 mm (Waters). The column was kept at 40 °C, and the flow rate was 0.4 mL/min. The mobile phases were (A) water with 0.1% formic acid and (B) acetonitrile containing 0.1% formic acid. The linear gradient was applied as follows: 0 min, 1% B; 1 min, 1% B; 16 min, 30% B; 21 min, 99% B; 22 min, 99% B; 25 min, 1% B. Mass spectrometric data were acquired using both positive (3.5 kv) and negative (−2.5 kv) modes over the range of m/z 100–1700. The drying gas temperature was 400 °C, the cone gas flow was 50 L/h, and the desolvation gas flow was 800 L/h. The sampling cone voltage was set to 30 V, and the source offset was 60 V. The accurate mass and composition of the precursor and fragment ions were calculated and sequenced using MassLynx4.1 software (Waters). One raw data set contained low‐energy data (6 eV, MS, precursor ions) and high‐energy data (15–45 eV, msE) for all of the fragment ions. Each sample was acquired three times at random. The data process was performed using the MarkerLynx application manager for Masslynx4.1 software. PCA was performed with MarkerLynx software (Waters). Based on the diversity in PCA and the good separation of groups, supervised OPLS‐DA was then used to extract maximum information from the data set and to isolate the metabolites responsible for differences between red (XYP3 and M4) and yellow (P6 and H3) petals. Potential biomarkers for grouping were identified by analysing the S‐plot, which was plotted using covariance (p) and correlation (pcorr).

## Author contributions

XG and ZL conceived of the study. ZZ developed the addition plants, WF prepared the plant materials and performed the experiments, DC and QP performed bioinformatics analysis, and DC and FL performed metabolomics analysis. WF, XG and ZL prepared the manuscript. All authors contributed to revising the manuscript. All authors read and approved the final manuscript.

## Supporting information


**Figure S1** Comparison of the FPKM values of the EBGs and LBGs in *Brassica napus* (H3 and M4) and *Orychophragmus violaceus* (OvW and OvP).
**Figure S2** Comparison of the FPKM values of the ABGs in the phenylpropanoid pathway in *Brassica napus* (H3 and M4) and *Orychophragmus violaceus* (OvW and OvP).
**Figure S3** Comparison of the FPKM values of the regulatory genes acting on the anthocyanin biosynthesis pathway in *Brassica napus* (H3 and M4) and *Orychophragmus violaceus* (OvW and OvP). Note that here, *PAP1* represents all homologs of *AtPAP1*,* AtPAP2*,* AtMYB113* and *AtMYB114* in *Brassica rapa* that have high sequence similarity.
**Figure S4** The sequence alignment of *PAP2* in each of the samples or species.
**Figure S5** Protein features encoded by *OvPAP2*.
**Figure S6** Phenotypes of the offspring (T_2_ and F_2_ individuals) of the *XY355::OvPAP2 Brassica napus* plant.
**Figure S7** Relative abundance and mass spectrum of one biomarker that is differentially accumulated in the red (M4 and XYP3) and yellow (H3 and P6) petals of *Brassica napus*.

**Table S1** Summary of alignment statistics from the RNA‐Seq of *Brassica napus* (H3 and M4) and *Orychophragmus violaceus* (OvW and OvP).
**Table S2** The expression levels of anthocyanin biosynthesis genes in *Brassica napus* (H3 and M4) and *Orychophragmus violaceus* (OvW and OvP).
**Table S4** The primers for gene cloning and vector construction.
**Table S5** Identified anthocyanin biomarkers that were differentially accumulated in the red and yellow petals of *Brassica napus*.
**Table S6** qRT‐PCR primers.

**Table S3** A comparison of the unigenes between M4 and *Orychophragmus violceus* annotated as anthocyanin biosynthesis genes.

## References

[pbi12777-bib-0001] Albert, N.W. , Lewis, D.H. , Zhang, H. , Schwinn, K.E. , Jameson, P.E. and Davies, K.M. (2011) Members of an R2R3‐MYB transcription factor family in *Petunia* are developmentally and environmentally regulated to control complex floral and vegetative pigmentation patterning. Plant J. 65, 771–784. 21235651 10.1111/j.1365-313X.2010.04465.x

[pbi12777-bib-0002] Anders, S. and Huber, W. (2010) Differential expression analysis for sequence count data. Genome Biol. 11, R106. 20979621 10.1186/gb-2010-11-10-r106 PMC3218662

[pbi12777-bib-0003] Bak, S. , Beisson, F. , Bishop, G. , Hamberger, B. , Hofer, R. , Paquette, S. and Werck‐Reichhart, D. (2011) Cytochromes p450. Arabidopsis Book, 9, e0144. 22303269 10.1199/tab.0144 PMC3268508

[pbi12777-bib-0004] Borevitz, J.O. , Xia, Y. , Blount, J. , Dixon, R.A. and Lamb, C. (2000) Activation tagging identifies a conserved MYB regulator of phenylpropanoid biosynthesis. Plant Cell, 12, 2383–2394. 11148285 10.1105/tpc.12.12.2383 PMC102225

[pbi12777-bib-0005] Brocard, I. , Charlot, F. , Teoule, E. and Guerche, P. (2001) Petal Specific Promoter and Method for Obtaining Plants Having (P). Patent: JP 2001517450‐A 309‐0CT‐2001. European: Institut National De La Recherche Agronomique.

[pbi12777-bib-0006] Butelli, E. , Licciardello, C. , Zhang, Y. , Liu, J. , Mackay, S. , Bailey, P. , Reforgiato‐Recupero, G. *et al*. (2012) Retrotransposons control fruit‐specific, cold‐dependent accumulation of anthocyanins in blood oranges. Plant Cell, 24, 1242–1255. 22427337 10.1105/tpc.111.095232 PMC3336134

[pbi12777-bib-0007] Chittka, L. and Waser, N.M. (1997) Why red flowers are not invisible to bees. Israel J. Plant Sci. 45, 169–183.

[pbi12777-bib-0008] Clough, S.J. and Bent, A.F. (1998) Floral dip: a simplified method for *Agrobacterium*‐mediated transformation of *Arabidopsis thaliana* . Plant J. 16, 735–743. 10069079 10.1046/j.1365-313x.1998.00343.x

[pbi12777-bib-0009] Cook, S.M. , Sandoz, J.C. , Martin, A.P. , Murray, D.A. , Poppy, G.M. and Williams, I.H. (2005) Could learning of pollen odours by honey bees (*Apis mellifera*) play a role in their foraging behaviour? Physiol. Entomol. 30, 164–174.

[pbi12777-bib-0010] Cook, S.M. , Skellern, M.P. , Doring, T.F. and Pickett, J.A. (2013) Red oilseed rape? The potential for manipulation of petal colour in control strategies for the pollen beetle (*Meligethes aeneus*). Arthropod Plant Interact. 7, 249–258.

[pbi12777-bib-0011] Ding, L. , Zhao, Z.G. , Ge, X.H. and Li, Z.Y. (2014) Different timing and spatial separation of parental chromosomes in intergeneric somatic hybrids between *Brassica napus* and *Orychophragmus violaceus* . Genet. Mol. Res. 13, 2611–2618. 24782049 10.4238/2014.April.8.3

[pbi12777-bib-0012] Döring, T.F. , Skellern, M. , Watts, N. and Cook, S.M. (2012) Colour choice behaviour in the pollen beetle *Meligethes aeneus* (Coleoptera: Nitidulidae). Physiol. Entomol. 37, 360–378.

[pbi12777-bib-0013] Feyissa, D.N. , Lovdal, T. , Olsen, K.M. , Slimestad, R. and Lillo, C. (2009) The endogenous *GL3*, but not *EGL3*, gene is necessary for anthocyanin accumulation as induced by nitrogen depletion in *Arabidopsis* rosette stage leaves. Planta, 230, 747–754. 19621239 10.1007/s00425-009-0978-3

[pbi12777-bib-0014] Fu, W.Q. , Zhao, Z.G. , Ge, X.H. , Ding, L. and Li, Z.Y. (2014) Anatomy and transcript profiling of gynoecium development in female sterile *Brassica napus* mediated by one alien chromosome from *Orychophragmus violaceus* . BMC Genom. 15, 61. 10.1186/1471-2164-15-61 PMC3930543 24456102

[pbi12777-bib-0015] Fu, D.H. , Jiang, L.Y. , Masons, A.S. , Xiao, M.L. , Zhu, L.R. , Li, L.Z. , Zhou, Q.H. *et al*. (2016) Research progress and strategies for multifunctional rapeseed: a case study of China. J. Integr. Agr. 15, 1673–1684.

[pbi12777-bib-0501] Glover, B.J. and Martin, C. (2012) Anthocyanins. Curr. Biol. 22, R147–150. 22401890 10.1016/j.cub.2012.01.021

[pbi12777-bib-0016] Gonzalez, A. , Zhao, M. , Leavitt, J.M. and Lloyd, A.M. (2008) Regulation of the anthocyanin biosynthetic pathway by the TTG1/bHLH/Myb transcriptional complex in Arabidopsis seedlings. Plant J. 53, 814–827. 18036197 10.1111/j.1365-313X.2007.03373.x

[pbi12777-bib-0502] Grotewold, E. (2006) The genetics and biochemistry of floral pigments. Annu. Rev. Plant Biol. 57, 761–780. 16669781 10.1146/annurev.arplant.57.032905.105248

[pbi12777-bib-0017] Guo, N. , Cheng, F. , Wu, J. , Liu, B. , Zheng, S. , Liang, J. and Wang, X. (2014) Anthocyanin biosynthetic genes in *Brassica rapa* . BMC Genom. 15, 426. 10.1186/1471-2164-15-426 PMC4072887 24893600

[pbi12777-bib-0018] Haas, B.J. , Papanicolaou, A. , Yassour, M. , Grabherr, M. , Blood, P.D. , Bowden, J. , Couger, M.B. *et al*. (2013) *De novo* transcript sequence reconstruction from RNA‐seq using the Trinity platform for reference generation and analysis. Nat. Protoc. 8, 1494–1512. 23845962 10.1038/nprot.2013.084 PMC3875132

[pbi12777-bib-0019] He, Q. , Zhang, Z. and Zhang, L. (2016) Anthocyanin accumulation, antioxidant ability and stability, and a transcriptional analysis of anthocyanin biosynthesis in purple heading Chinese cabbage (*Brassica rapa* L. ssp. *pekinensis*). J. Agric. Food Chem. 64, 132–145. 26709726 10.1021/acs.jafc.5b04674

[pbi12777-bib-0020] Heim, M.A. , Jakoby, M. , Werber, M. , Martin, C. , Weisshaar, B. and Bailey, P.C. (2003) The basic helix‐loop‐helix transcription factor family in plants: a genome‐wide study of protein structure and functional diversity. Mol. Biol. Evol. 20, 735–747. 12679534 10.1093/molbev/msg088

[pbi12777-bib-0021] Jing, J. , Shi, Y. , Zhang, Q. , Wang, J. and Ruan, J. (2017) Prediction of Chinese green tea ranking by metabolite profiling using ultra‐performance liquid chromatography‐quadrupole time‐of‐flight mass spectrometry (UPLC‐Q‐TOF/MS). Food Chem. 221, 311–316. 27979208 10.1016/j.foodchem.2016.10.068

[pbi12777-bib-0022] Kui, L.W. , Bolitho, K. , Grafton, K. , Kortstee, A. , Karunairetnam, S. , McGhie, T.K. , Espley, R.V. *et al*. (2010) An R2R3 MYB transcription factor associated with regulation of the anthocyanin biosynthetic pathway in Rosaceae. BMC Plant Biol. 10, 50. 20302676 10.1186/1471-2229-10-50 PMC2923524

[pbi12777-bib-0023] Mänd, M. , Williams, I.H. , Viik, E. and Karise, R. (2010) Oilseed rape, bees and integrated pest management. In Biocontrol‐Based Integrated Management of Oilseed Rape Pests ( Williams, I.H. , ed.), pp. 357–379. London: Springer‐Verlag.

[pbi12777-bib-0024] Menzel, R. (1985) Learning in honey bees in an ecological and behavioural context. Fort. Zool. 31, 55–74.

[pbi12777-bib-0025] Mushtaq, M.A. , Pan, Q. , Chen, D. , Zhang, Q. , Ge, X. and Li, Z. (2016) Comparative leaves transcriptome analysis emphasizing on accumulation of anthocyanins in *Brassica*: molecular regulation and potential interaction with photosynthesis. Front. Plant Sci. 7, 311. 27047501 10.3389/fpls.2016.00311 PMC4796009

[pbi12777-bib-0026] Nishihara, M. and Nakatsuka, T. (2011) Genetic engineering of flavonoid pigments to modify flower color in floricultural plants. Biotechnol. Lett. 33, 433–441. 21053046 10.1007/s10529-010-0461-z

[pbi12777-bib-0027] Noda, N. , Aida, R. , Kishimoto, S. , Ishiguro, K. , Fukuchi‐Mizutani, M. , Tanaka, Y. and Ohmiya, A. (2013) Genetic engineering of novel bluer‐colored chrysanthemums produced by accumulation of delphinidin‐based anthocyanins. Plant Cell Physiol. 54, 1684–1695. 23926063 10.1093/pcp/pct111

[pbi12777-bib-0028] Nukumizu, Y. , Wada, T. and Tominaga‐Wada, R. (2013) Tomato (*Solanum lycopersicum*) homologs of TRIPTYCHON (SlTRY) and GLABRA3 (SlGL3) are involved in anthocyanin accumulation. Plant Signal. Behav. 8, e24575. 23603939 10.4161/psb.24575 PMC3907391

[pbi12777-bib-0029] Petroni, K. and Tonelli, C. (2011) Recent advances on the regulation of anthocyanin synthesis in reproductive organs. Plant Sci. 181, 219–229. 21763532 10.1016/j.plantsci.2011.05.009

[pbi12777-bib-0030] Ramsay, N.A. and Glover, B.J. (2005) MYB‐bHLH‐WD40 protein complex and the evolution of cellular diversity. Trends Plant Sci. 10, 63–70. 15708343 10.1016/j.tplants.2004.12.011

[pbi12777-bib-0031] Robinson, M.D. , McCarthy, D.J. and Smyth, G.K. (2010) edgeR: a bioconductor package for differential expression analysis of digital gene expression data. Bioinformatics, 26, 139–140. 19910308 10.1093/bioinformatics/btp616 PMC2796818

[pbi12777-bib-0032] Seitz, C. , Eder, C. , Deiml, B. , Kellner, S. , Martens, S. and Forkmann, G. (2006) Cloning, functional identification and sequence analysis of flavonoid 3′‐hydroxylase and flavonoid 3′,5′‐hydroxylase cDNAs reveals independent evolution of flavonoid 3′,5′‐hydroxylase in the Asteraceae family. Plant Mol. Biol. 61, 365–381. 16830174 10.1007/s11103-006-0012-0

[pbi12777-bib-0033] Sobel, J.M. and Streisfeld, M.A. (2013) Flower color as a model system for studies of plant evo‐devo. Front. Plant Sci. 4, 321. 23970892 10.3389/fpls.2013.00321 PMC3748380

[pbi12777-bib-0034] Stracke, R. , Werber, M. and Weisshaar, B. (2001) The R2R3‐MYB gene family in *Arabidopsis thaliana* . Curr. Opin. Plant Biol. 4, 447–456. 11597504 10.1016/s1369-5266(00)00199-0

[pbi12777-bib-0035] Sun, J. , Xiao, Z. , Lin, L.Z. , Lester, G.E. , Wang, Q. , Harnly, J.M. and Chen, P. (2013) Profiling polyphenols in five *Brassica* species microgreens by UHPLC‐PDA‐ESI/HRMS(n.). J. Agric. Food Chem. 61, 10960–10970. 24144328 10.1021/jf401802n PMC3915300

[pbi12777-bib-0036] Tamura, K. , Stecher, G. , Peterson, D. , Filipski, A. and Kumar, S. (2013) MEGA6: molecular evolutionary genetics analysis version 6.0. Mol. Biol. Evol. 30, 2725–2729. 24132122 10.1093/molbev/mst197 PMC3840312

[pbi12777-bib-0037] Tanaka, Y. and Brugliera, F. (2013) Flower colour and cytochromes P450. Philos. Trans. R. Soc. Lond. B Biol. Sci. 368, 20120432. 23297355 10.1098/rstb.2012.0432 PMC3538422

[pbi12777-bib-0038] Tanaka, Y. , Brugliera, F. , Kalc, G. , Senior, M. , Dyson, B. , Nakamura, N. , Katsumoto, Y. *et al*. (2010) Flower color modification by engineering of the flavonoid biosynthetic pathway: practical perspectives. Biosci. Biotechnol. Biochem. 74, 1760–1769. 20834175 10.1271/bbb.100358

[pbi12777-bib-0503] Tanaka, Y. , Sasaki, N. and Ohmiya, A. (2008) Biosynthesis of plant pigments: anthocyanins, betalains and carotenoids. Plant J. 54, 733–749. 18476875 10.1111/j.1365-313X.2008.03447.x

[pbi12777-bib-0039] Trapnell, C. , Roberts, A. , Goff, L. , Pertea, G. , Kim, D. , Kelley, D.R. , Pimentel, H. *et al*. (2012) Differential gene and transcript expression analysis of RNA‐seq experiments with TopHat and Cufflinks. Nat. Protoc. 7, 562–578. 22383036 10.1038/nprot.2012.016 PMC3334321

[pbi12777-bib-0040] U N . (1935) Genome analysis in *Brassica* with special reference to the experimental formation of *B. napus* and peculiar mode of fertilization. Jap. J. Bot. 7, 389–452.

[pbi12777-bib-0041] de Vetten, N. , Wolters, A.M. , Raemakers, K. , van der Meer, I. , ter Stege, R. , Heeres, E. , Heeres, P. *et al*. (2003) A transformation method for obtaining marker‐free plants of a cross‐pollinating and vegetatively propagated crop. Nat. Biotechnol. 21, 439–442. 12627169 10.1038/nbt801

[pbi12777-bib-0042] Wessinger, C.A. and Rausher, M.D. (2012) Lessons from flower colour evolution on targets of selection. J. Exp. Bot. 63, 5741–5749. 23048126 10.1093/jxb/ers267

[pbi12777-bib-0504] Winkel‐Shirley, B. (2001) Flavonoid biosynthesis. A colorful model for genetics, biochemistry, cell biology, and biotechnology. Plant Physiol. 126, 485–493. 11402179 10.1104/pp.126.2.485 PMC1540115

[pbi12777-bib-0043] Wu, X. and Prior, R.L. (2005) Identification and characterization of anthocyanins by high‐performance liquid chromatography‐electrospray ionization‐tandem mass spectrometry in common foods in the United States: vegetables, nuts, and grains. J. Agric. Food Chem. 53, 3101–3113. 15826066 10.1021/jf0478861

[pbi12777-bib-0044] Xia, S.Q. , Wang, Z.X. , Zhang, H.Y. , Hu, K.N. , Zhang, Z.Q. , Qin, M.M. , Dun, X.L. *et al*. (2016) Altered transcription and neofunctionalization of duplicated genes rescue the harmful effects of a chimeric gene in *Brassica napus* . Plant Cell, 28, 2060–2078. 27559024 10.1105/tpc.16.00281 PMC5059798

[pbi12777-bib-0045] Zhang, Y. (2014) Research on the problems and development strategies of Wuyuan ecotourism. Marketing Res. 6, 41–42. (in Chinese).

[pbi12777-bib-0046] Zhang, Y. , Butelli, E. and Martin, C. (2014a) Engineering anthocyanin biosynthesis in plants. Curr. Opin. Plant Biol. 19, 81–90. 24907528 10.1016/j.pbi.2014.05.011

[pbi12777-bib-0047] Zhang, Y. , Chen, G. , Dong, T. , Pan, Y. , Zhao, Z. , Tian, S. and Hu, Z. (2014b) Anthocyanin accumulation and transcriptional regulation of anthocyanin biosynthesis in purple bok choy (*Brassica rapa* var. chinensis). J. Agric. Food Chem. 62, 12366–12376. 25419600 10.1021/jf503453e

[pbi12777-bib-0048] Zhao, Z.G. , Hu, T.T. , Ge, X.H. , Du, X.Z. , Ding, L. and Li, Z.Y. (2008) Production and characterization of intergeneric somatic hybrids between *Brassica napus* and *Orychophragmus violaceus* and their backcrossing progenies. Plant Cell Rep. 27, 1611–1621. 18626647 10.1007/s00299-008-0582-1

[pbi12777-bib-0049] Zhu, C. , Bai, C. , Sanahuja, G. , Yuan, D. , Farre, G. , Naqvi, S. , Shi, L. *et al*. (2010) The regulation of carotenoid pigmentation in flowers. Arch. Biochem. Biophys. 504, 132–141. 20688043 10.1016/j.abb.2010.07.028

[pbi12777-bib-0050] Zimmermann, I.M. , Heim, M.A. , Weisshaar, B. and Uhrig, J.F. (2004) Comprehensive identification of *Arabidopsis thaliana* MYB transcription factors interacting with R/B‐like BHLH proteins. Plant J. 40, 22–34. 15361138 10.1111/j.1365-313X.2004.02183.x

