# Supplementary material for: Production of red‐flowered oilseed rape via the ectopic expression of Orychophragmus violaceus OvPAP2
Source: Plant Biotechnol J. 2017 Jul 26;16(2):367–80. doi: 10.1111/pbi.12777 (PMC5787836; doi:10.1111/pbi.12777)
Supplement: Supplementary file 1 — Figure S1 Comparison of the FPKM values of the EBGs and LBGs in Brassica napus (H3 and M4) and Orychophragmus violaceus (OvW and OvP). Figure S2 Comparison of the FPKM values of the ABGs in the phenylpropanoid pathway in Brassica napus (H3 and M4) and Orychophragmus violaceus (OvW and OvP). Figure S3 Comparison of the FPKM values of the regulatory genes acting on the anthocyanin biosynthesis pathway in Brassica napus (H3 and M4) and Orychophragmus violaceus (OvW and OvP). Note that here, PAP1 represents all homologs of AtPAP1, AtPAP2, AtMYB113 and AtMYB114 in Brassica rapa that have high sequence similarity. Figure S4 The sequence alignment of PAP2 in each of the samples or species. Figure S5 Protein features encoded by OvPAP2. Figure S6 Phenotypes of the offspring (T2 and F2 individuals) of the XY355::OvPAP2 Brassica napus plant. Figure S7 Relative abundance and mass spectrum of one biomarker that is differentially accumulated in the red (M4 and XYP3) and yellow (H3 and P6) petals of Brassica napus. [file PBI-16-367-s002.docx]

**
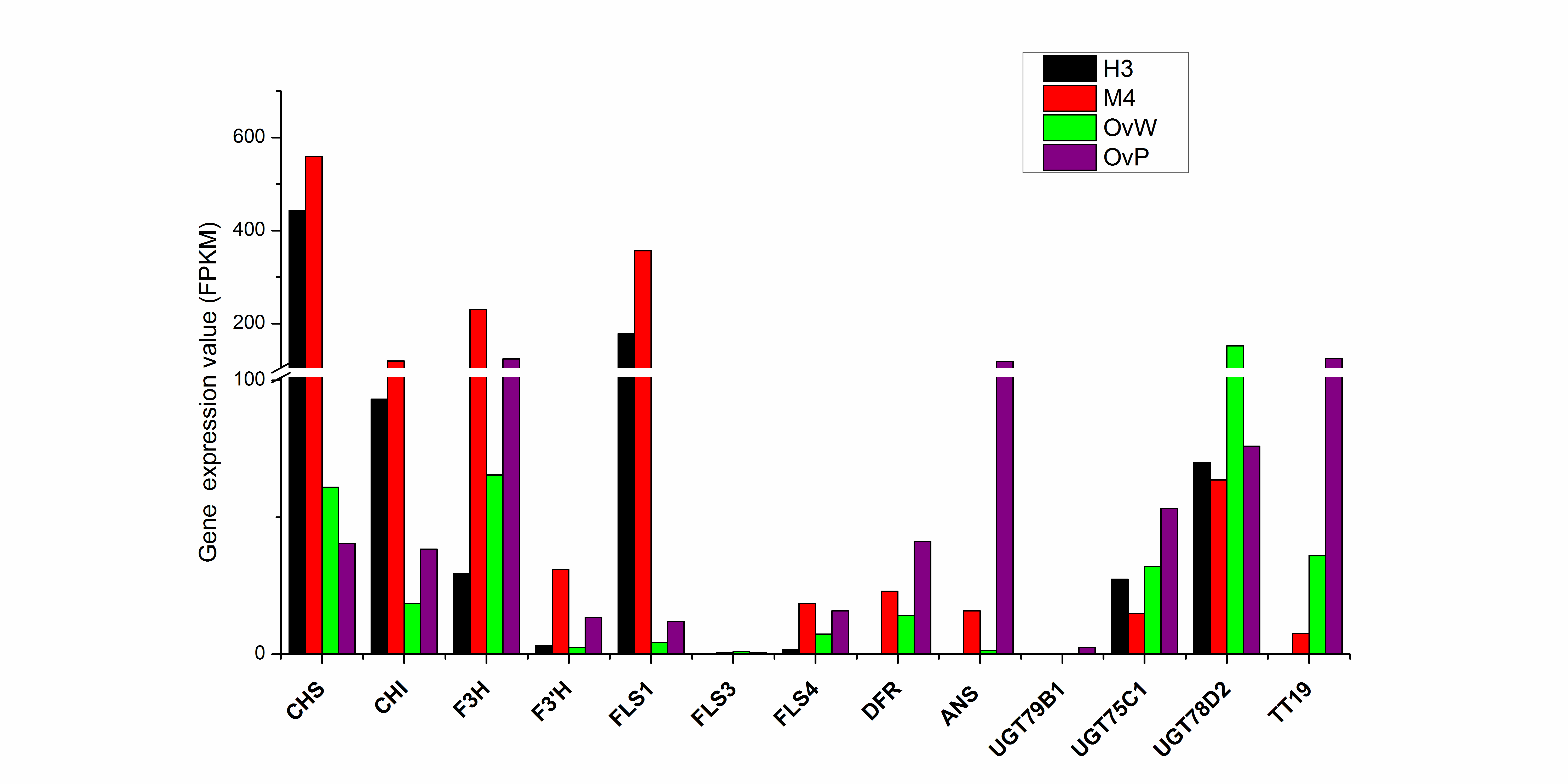
Figure S1** Comparison of the FPKM values of the EBGs and LBGs in *Brassica napus* (H3 and M4) and *Orychophragmus violaceus* (OvW and OvP). FPKM: fragments per kilobase of transcript sequence per million base pairs sequenced; EBGs: early biosynthesis genes; LBGs: late biosynthesis genes.

**
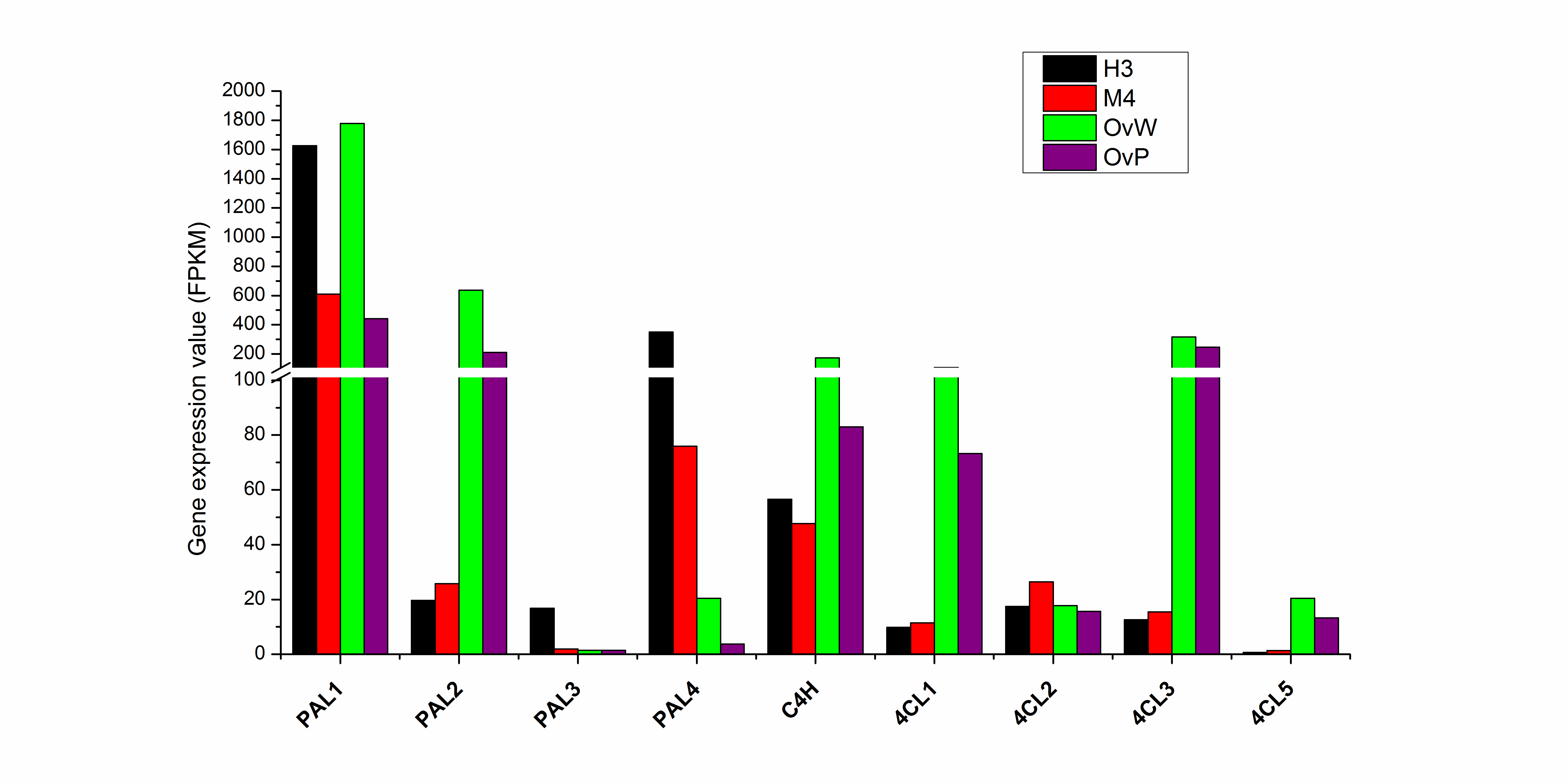
**

**Figure S2** Comparison of the FPKM values of the ABGs in the phenylpropanoid pathway in *Brassica napus* (H3 and M4) and *Orychophragmus violaceus* (OvW and OvP). FPKM: fragments per kilobase of transcript sequence per million base pairs sequenced; ABGs: anthocyanin biosynthesis genes.

**
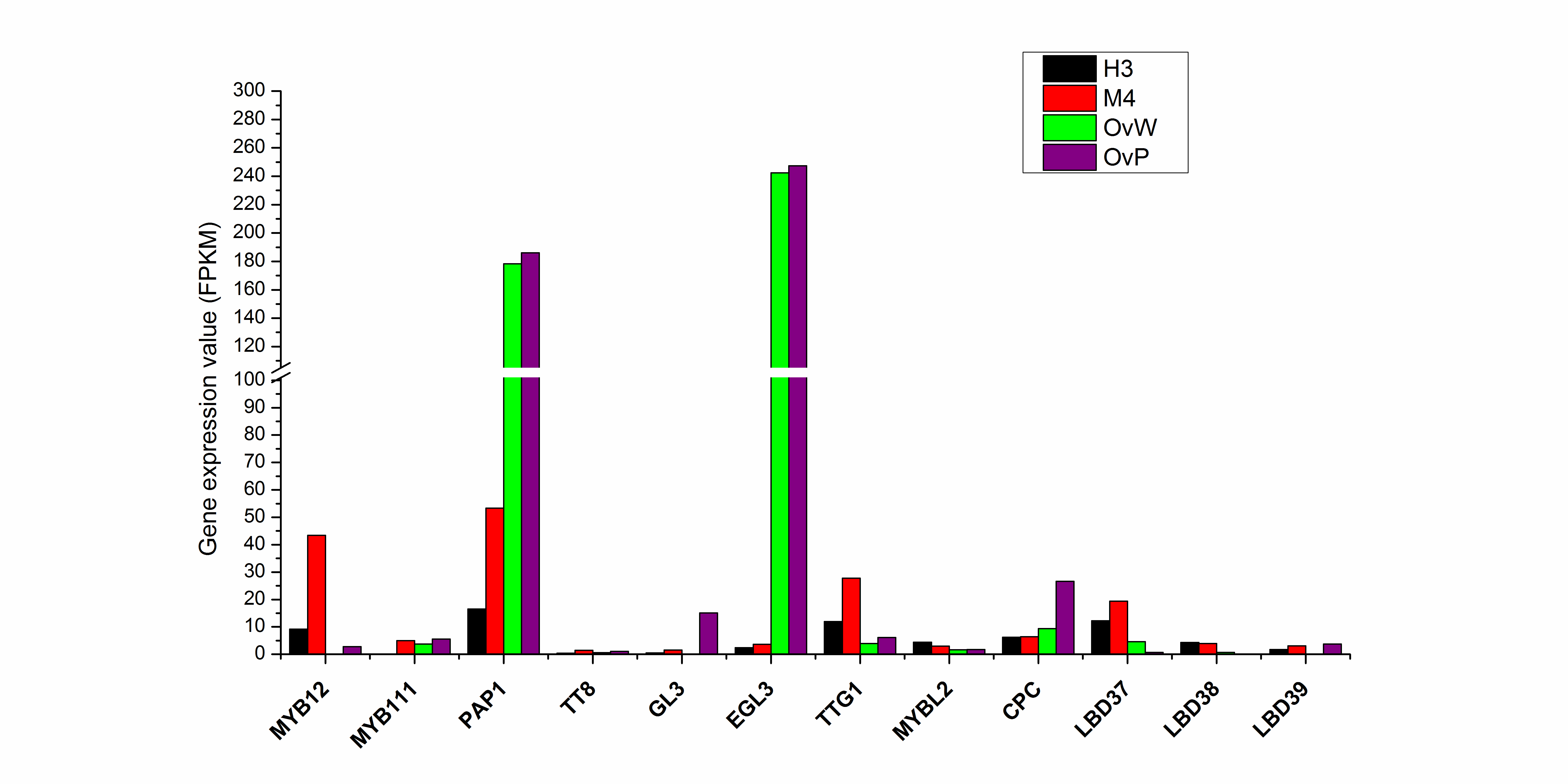
**

**Figure S3** Comparison of the FPKM values of the regulatory genes acting on the anthocyanin biosynthesis pathway in *Brassica napus* (H3 and M4) and *Orychophragmus violaceus* (OvW and OvP). Note that here, *PAP1* represents all homologs of *AtPAP1*, *AtPAP2*, *AtMYB113* and *AtMYB114* in *Brassica rapa* that have high sequence similarity. FPKM: fragments per kilobase of transcript sequence per million base pairs sequenced.

**
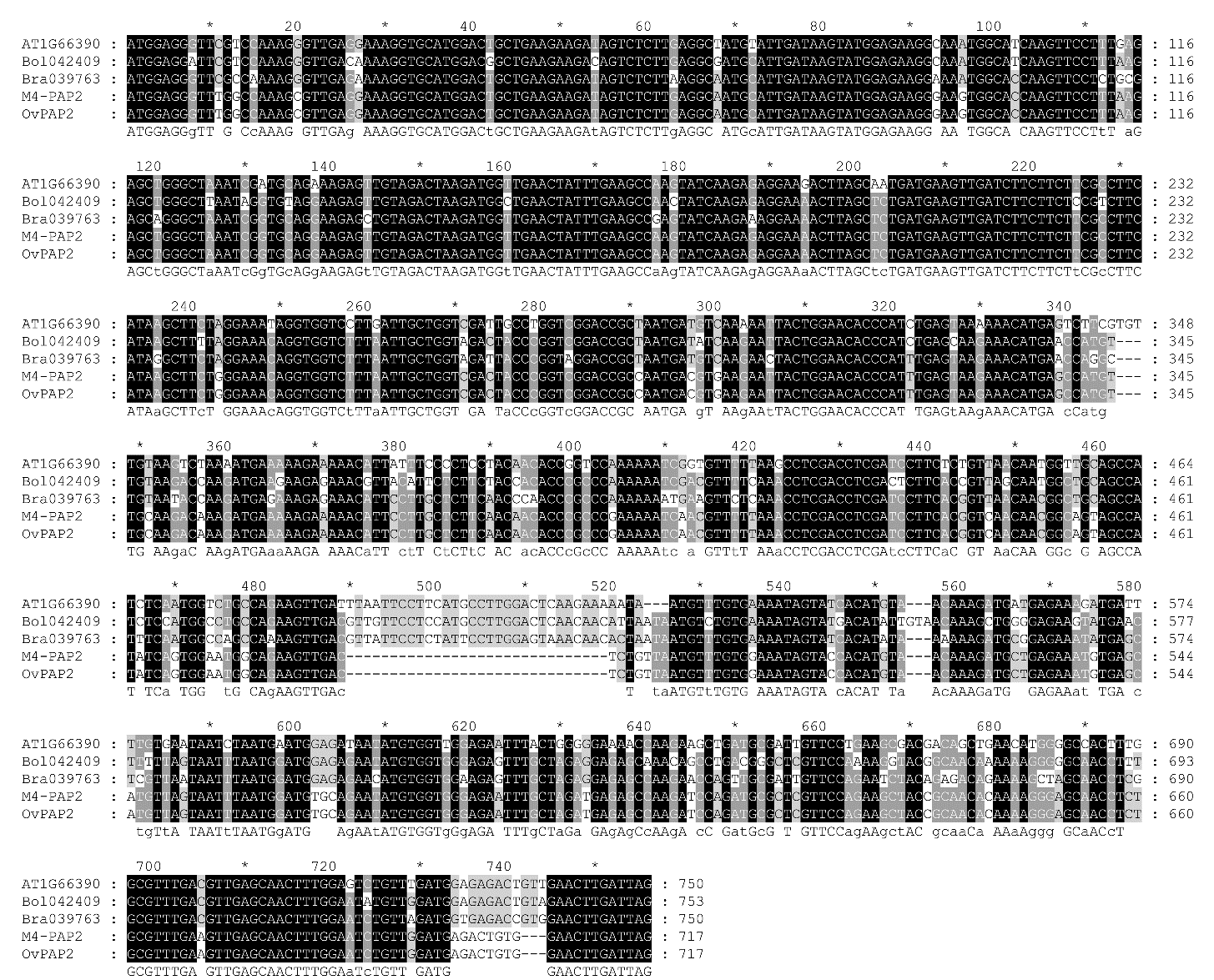
**

**Figure S4** The sequence alignment of *PAP2* in each of the samples or species.

**
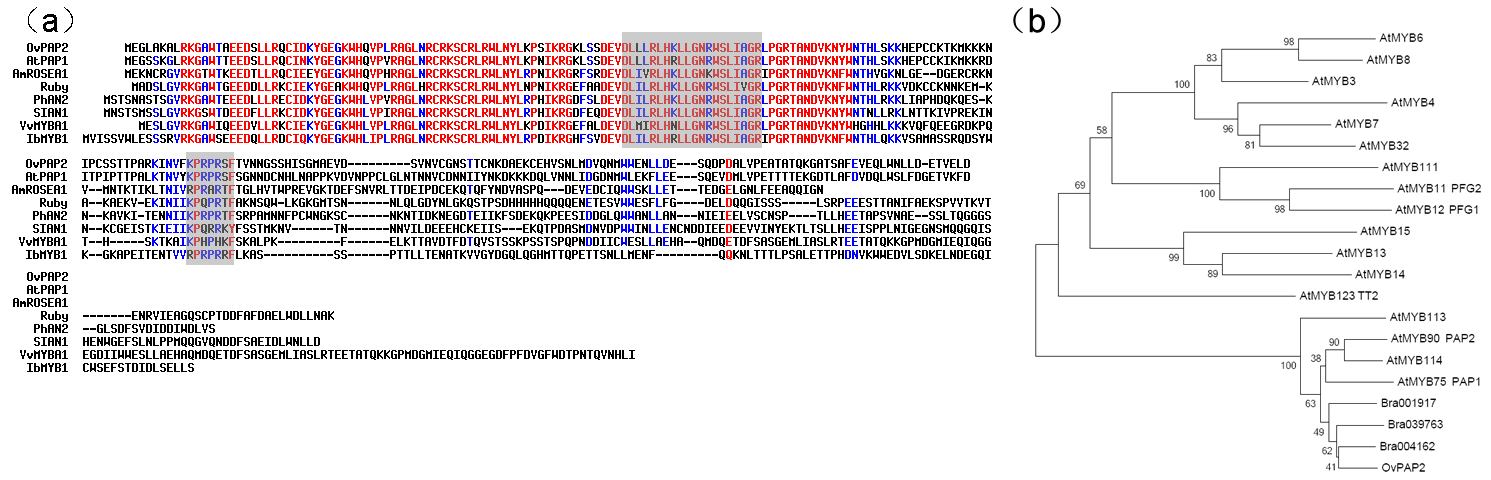
**

**Figure S5** Protein features encoded by *OvPAP2*. (a) Protein alignment of OvPAP2 with the characterized members of the anthocyanin-specific family of MYB factors. Identical amino acids are shown in pink. The GenBank accession numbers of these proteins are as follows: PhAN2 (Petunia3hybrida), AAF66727.1; Sl AN1 (tomato [*Solanum lycopersicum*]), AAQ55181.1; Ruby (*Citrus sinensis*), AFB73909.1; VvMYBA1 (*Vitis vinifera*), ABB87013.1; IbMYB1 (*Ipomoea batatas*), BAF45118.1; At PAP1 (*Arabidopsis*), ABB03877.1; and AmROSEA1 (snapdragon [*Antirrhinum majus* L.]), ABB83826.1. The signature motif within the R3 MYB DNA binding domain for interaction with bHLH proteins and the conserved motif defining members of R2R3 MYB subgroup 6 are boxed in gray based on Butelli *et al.*, (2012). (b) Phylogenetic analysis showing that the OvPAP2 transcription factor is clustered with anthocyanin-specific members of subgroup 6 from the R2R3 MYB family in *Arabidopsis*. Locus identifiers for *Arabidopsis* proteins are AtMYB3 (At1g22640), AtMYB4 (At4g38620), AtMYB6 (At4g09460), AtMYB7 (At2g16720), AtMYB8 (At1g35515), AtMYB13 (At1g06180), AtMYB14 (At2g31180), AtMYB15 (At3g23250), AtMYB32 (At4g34990), AtMYB11 (At3g62610), AtMYB12 (At2g47460), AtMYB111 (At5g49330), AtMYB113 (At1g66370), AtMYB114 (At1g66380), AtMYB75 (At1g56650), AtMYB90 (At1g66390), and AtMYB123 (At5g35550).


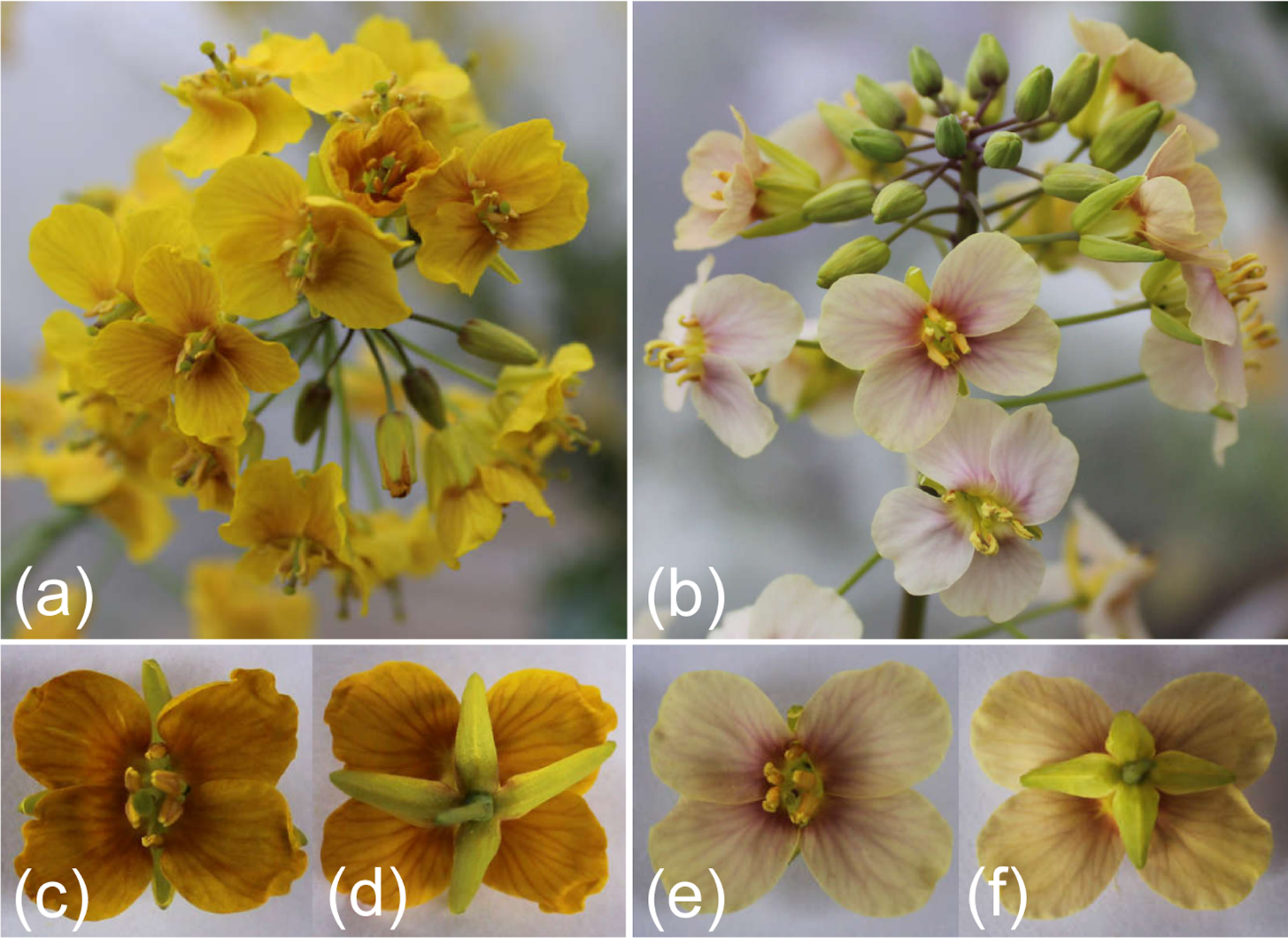


**Figure S6** Phenotypes of the offspring (T_2_ and F_2_ individuals) of the *XY355::OvPAP2* *Brassica napus* plant. (a) and (b) Inflorescence. (c) and (e) Adaxial side of flowers. (d) and (f) Abaxial side of flowers. (a), (c) and (d) T_2_ individual *XY355::OvPAP2* plant. (b), (e) and (f) F_2_ individual from a cross between the *XY355::OvPAP2* plant and *Brassica napus* cv. G1300, which has white flowers.


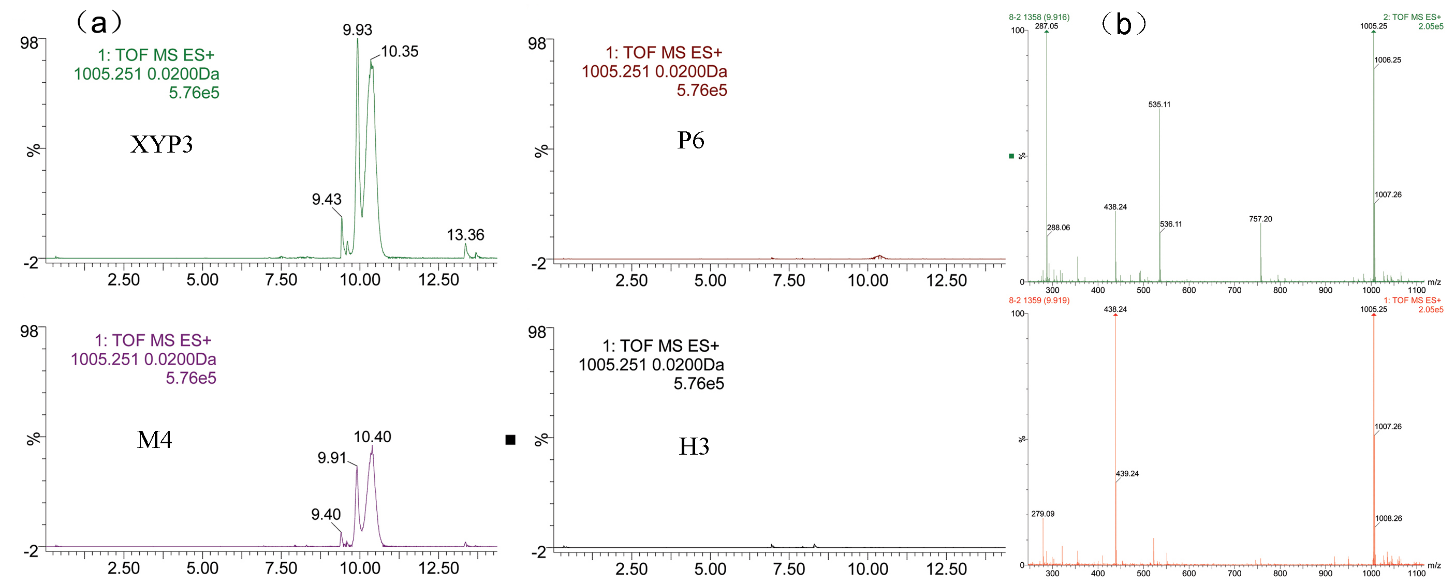
**Figure S7** Relative abundance and mass spectrum of one biomarker that is differentially accumulated in the red (M4 and XYP3) and yellow (H3 and P6) petals of *Brassica napus*. (a) Extraction peaks at a molecular weight of 1005.2124 in different samples. (b) First and second mass spectra of the extracted peak. XYP3: *XY355::OvPAP2* plant; P6: *CaMV35S::OvPAP2* plant with red anthers.
