# Supplementary material for: Production of red‐flowered oilseed rape via the ectopic expression of Orychophragmus violaceus OvPAP2
Source: Plant Biotechnol J. 2017 Jul 26;16(2):367–80. doi: 10.1111/pbi.12777 (PMC5787836; doi:10.1111/pbi.12777)
Supplement: Supplementary file 2 — Table S1 Summary of alignment statistics from the RNA‐Seq of Brassica napus (H3 and M4) and Orychophragmus violaceus (OvW and OvP). Table S2 The expression levels of anthocyanin biosynthesis genes in Brassica napus (H3 and M4) and Orychophragmus violaceus (OvW and OvP). Table S4 The primers for gene cloning and vector construction. Table S5 Identified anthocyanin biomarkers that were differentially accumulated in the red and yellow petals of Brassica napus. Table S6 qRT‐PCR primers. [file PBI-16-367-s001.docx]

| **Sample** | **Raw data** | **Clean reads** | **Total mapped** | **Unique mapped** | **Expressed genes** |
| --- | --- | --- | --- | --- | --- |
| M4 | 33814714 | 30275646 (89.53%) | 17296611 (57.13%) | 16124412 (93.22%) | 31956 (77.90%) |
| H3 | 34056222 | 30383979 (89.22%) | 18118720 (59.63%) | 16941644 (93.50%) | 30979 (75.52%) |
| OvP | 29536508 | 26080543 (88.30%) | 2715153 (10.41%) | 1888900 (69.57%) | 30009 (73.16%) |
| OvW | 31797672 | 28414928 (89.36%) | 2943819 (10.36%) | 2075972 (70.52%) | 30275 (73.81%) |

**Table S1** Summary of alignment statistics from the RNA-Seq of *Brassica napus* (H3 and M4) and *Orychophragmus violaceus* (OvW and OvP)

**Table S2** The expression levels of anthocyanin biosynthesis genes in *Brassica napus* (H3 and M4) and *Orychophragmus violaceus* (OvW and OvP)

| **Gene ID** | **H3** | **M4** | **OvW** | **OvP** | **Tair** | **Gene symbol** |
| --- | --- | --- | --- | --- | --- | --- |
| Bra005221 | 1562.34 | 562.47 | 1251.3 | 260.91 | AT2G37040 | PAL1 |
| Bra017210 | 64.81 | 47.2 | 527.9 | 181.11 | AT2G37040 | PAL1 |
| Bra006985 | 10.35 | 19.49 | 204 | 54.16 | AT3G53260 | PAL2 |
| Bra039777 | 4.93 | 1.71 | 178.48 | 68.72 | AT3G53260 | PAL2 |
| Bra003126 | 4.36 | 4.53 | 255.14 | 87.74 | AT3G53260 | PAL2 |
| Bra028793 | 16.84 | 1.96 | 0.54 | 1.2 | AT5G04230 | PAL3 |
| Bra030322 | 0 | 0 | 0.92 | 0.25 | AT5G04230 | PAL3 |
| Bra029831 | 351.38 | 75.95 | 20.42 | 3.74 | AT3G10340 | PAL4 |
| Bra018311 | 51.1 | 35.19 | 49.87 | 26.5 | AT2G30490 | C4H |
| Bra021636 | 0.31 | 0.44 | 53.63 | 16.04 | AT2G30490 | C4H |
| Bra021637 | 3.36 | 6.46 | 4.16 | 15.35 | AT2G30490 | C4H |
| Bra022802 | 0.08 | 0.74 | 3.75 | 4.9 | AT2G30490 | C4H |
| Bra022803 | 1.74 | 4.88 | 61.27 | 20.23 | AT2G30490 | C4H |
| Bra030429 | 9.87 | 11.47 | 106.36 | 73.32 | AT1G51680 | 4CL1 |
| Bra031262 | 0.16 | 0.14 | 1.58 | 2.89 | AT3G21240 | 4CL2 |
| Bra031263 | 0.92 | 1.05 | 4.25 | 6.05 | AT3G21240 | 4CL2 |
| Bra031265 | 0 | 0 | 3.25 | 1.26 | AT3G21240 | 4CL2 |
| Bra031266 | 16.35 | 25.25 | 8.71 | 5.42 | AT3G21240 | 4CL2 |
| Bra004109 | 12.65 | 15.44 | 317.32 | 246.08 | AT1G65060 | 4CL3 |
| Bra001819 | 0.3 | 1.26 | 6.16 | 2.92 | AT3G21230 | 4CL5 |
| Bra001820 | 0.37 | 0.14 | 14.23 | 10.38 | AT3G21230 | 4CL5 |
| Bra008792 | 57.2 | 109.27 | 2.84 | 3.12 | AT5G13930 | CHS |
| Bra006224 | 131.18 | 86.76 | 17.8 | 21.34 | AT5G13930 | CHS |
| Bra023441 | 253.25 | 361.94 | 6.1 | 5.3 | AT5G13930 | CHS |
| Bra036307 | 1 | 1.52 | 32.59 | 9.85 | AT5G13930 | CHS |
| Bra020688 | 0 | 0 | 1.63 | 0.89 | AT5G13930 | CHS |
| Bra007142 | 61.22 | 96.66 | 12.74 | 32.21 | AT3G55120 | CHI |
| Bra003209 | 27.33 | 22.71 | 4.27 | 6.19 | AT3G55120 | CHI |
| Bra017728 | 4.58 | 0.63 | 1.62 | 0 | AT3G55120 | CHI |
| Bra036828 | 28.43 | 221.52 | 38.02 | 84.98 | AT3G51240 | F3H |
| Bra029996 | 0.05 | 0.06 | 0 | 2.42 | AT3G51240 | F3H |
| Bra012862 | 0.84 | 9.01 | 27.45 | 36.7 | AT3G51240 | F3H |
| Bra009312 | 3.19 | 30.88 | 2.51 | 13.44 | AT5G07990 | F3'H |
| Bra009358 | 178.34 | 357.05 | 4.29 | 12.04 | AT5G08640 | FLS1 |
| Bra038647 | 0.13 | 0 | 0 | 0 | AT5G63590 | FLS3 |
| Bra029211 | 0 | 0.54 | 1.03 | 0.57 | AT5G63590 | FLS3 |
| Bra037747 | 0 | 0.13 | 0 | 0 | AT5G63590 | FLS3 |
| Bra038648 | 1.52 | 1.09 | 7 | 15.4 | AT5G63595 | FLS4 |
| Bra029212 | 0.25 | 17.4 | 0.38 | 0.42 | AT5G63595 | FLS4 |
| Bra027457 | 0.25 | 22.97 | 14.14 | 41.15 | AT5G42800 | DFR |
| Bra013652 | 0 | 13.51 | 0.89 | 69.3 | AT4G22880 | ANS |
| Bra019350 | 0 | 2.37 | 0.45 | 50 | AT4G22880 | ANS |
| Bra003021 | 0 | 0 | 0 | 0.83 | AT5G54060 | UGT79B1 |
| Bra035004 | 0 | 0.1 | 0 | 1.66 | AT5G54060 | UGT79B1 |
| Bra038445 | 27.4 | 14.93 | 32.11 | 53.13 | AT4G14090 | UGT75C1 |
| Bra023594 | 70.06 | 63.63 | 152.54 | 75.96 | AT5G17050 | UGT78D2 |
| Bra004456 | 5.43 | 40.14 | 0 | 0.96 | AT2G47460 | MYB12 |
| Bra000453 | 3.77 | 3.26 | 0 | 1.86 | AT2G47460 | MYB12 |
| Bra037419 | 0 | 4.7 | 0 | 2.05 | AT5G49330 | MYB111 |
| Bra020647 | 0 | 0 | 1.02 | 0 | AT5G49330 | MYB111 |
| Bra036145 | 0.11 | 0.3 | 2.77 | 3.55 | AT5G49330 | MYB111 |
| Bra001917  Bra004162  Bra039763 | 0.26  11.74  4.49 | 7.61  43.68  2.07 | 26.98  144.38  7.06 | 33.18  142.31  10.59 | AT1G56650/ AT1G66390/ AT1G66370/ AT1G66380 | PAP1/ PAP2/ MYB113/ MYB114 |
| Bra037887 | 0.39 | 1.43 | 0.64 | 1.06 | AT4G09820 | TT8 |
| Bra025508 | 0.53 | 1.52 | 0 | 15.13 | AT5G41315 | GL3 |
| Bra027796 | 2.34 | 2.71 | 54.33 | 40.01 | AT1G63650 | EGL3 |
| Bra027653 | 0.03 | 0.9 | 188.14 | 207.31 | AT1G63650 | EGL3 |
| Bra009770 | 11.9 | 27.77 | 2.63 | 4.7 | AT5G24520 | TTG1 |
| Bra029411 | 0 | 0 | 1.33 | 1.46 | AT5G24520 | TTG1 |
| Bra016164 | 3.12 | 1.53 | 0.82 | 1.7 | AT1G71030 | MYBL2 |
| Bra007957 | 1.3 | 1.42 | 0.84 | 0 | AT1G71030 | MYBL2 |
| Bra004539 | 6.21 | 6.46 | 9.34 | 26.68 | AT2G46410 | CPC |
| Bra039283 | - | - | - | - | AT2G46410 | CPC |
| Bra012164 | 9.08 | 16.29 | 1.25 | 0.69 | AT5G67420 | LBD37 |
|  |  |  |  |  |  |  |
| Bra031833 | 2.34 | 0.94 | 1.33 | 0 | AT5G67420 | LBD37 |
| Bra037847 | 0.83 | 2.13 | 2.03 | 0 | AT5G67420 | LBD37 |
| Bra036040 | 0.24 | 0.68 | 0.65 | 0 | AT3G49940 | LBD38 |
| Bra012913 | 4.03 | 3.21 | 0 | 0 | AT3G49940 | LBD38 |
| Bra011772 | 0.97 | 2.68 | 0 | 3.71 | AT4G37540 | LBD39 |
| Bra017831 | 0.78 | 0.44 | 0 | 0 | AT4G37540 | LBD39 |
| Bra008570 | 0.09 | 1.84 | 34.51 | 122.04 | AT5G17220 | TT19 |
| Bra023602 | 0 | 5.74 | 1.49 | 3.27 | AT5G17220 | TT19 |

Note: numerals underlined represents that the gene was differential expressed gene (DEG) in the corresponding group of samples.

**Table S4** The primers for gene cloning and vector construction

| **Primers** | **Sequences** |
| --- | --- |
| PAP2-1F | GTAGCTATCGTTGGTCCATGG |
| PAP2-1R | GCACACACAAACAAACAGTCG |
| PAP2-XF | GCTCTAGAGTAGCTATCGTTGGTCCATG |
| PAP2-BR | CGGGATCCCACTAATCAAGTTCCACAGTCTC |
| XY355-HF | CCCAAGCTTGTCGACTAAATTGAAACAGAAAGCCACA |
| XY355-PR | AACTGCAGTTGTTTTTTCTTTTAAATTGTAGTGAG |
| PAP2-PF | AACTGCAGATGGAGGGTTTGGCCAAAGCGTTGAGGAAAG |
| PAP2-KR | GGGGTACCCTAATCAAGTTCCACAGTCTCATCCAACAG |
| RV-M | GAGCGGATAACAATTTCACACAGG |
| M13-47 | CGCCAGGGTTTTCCCAGTCACGAC |
| pMDC83-nos | TCGCAAGACCGGCAACAGGA |

**Table S5** Identified anthocyanin biomarkers that were differentially accumulated in the red and yellow petals of *Brassica napus*

| Abounding in red petals | | | | |  |
| --- | --- | --- | --- | --- | --- |
| No. | Ret. Time | [M + M]^+^ m/z | MS/MS (m/z) | Tentative identification | Reference |
| 1 | 6.4351 | 789.2081 | 627/465/303 | Delphinidin-3 caffeoylglucoside -5-glucoside | 1 |
| 2 | 6.9557 | 859.2138 | 611/ 535/287 | Cyanidin 3-diglucoside-5-malonlyglucoside | 1,2,5 |
| 3 | 8.0913 | 697.1621 | 551/449/287 | Cyanidin-3-glucoside-5- malonlyglucoside | 1 |
| 4 | 8.189 | 627.1564 | 465/303 | Delphinidin-3,5-glucoside | 1 |
| 5* | 8.6855 | 713.1562 | 551/465/303 | Delphinidin -3- malonyl glucoside-5-glucoside | - |
| 6 | 8.8719 | 697.1609 | 551/449/287 | Cyanidin-3-glucoside-5- malonlyglucosid | 1 |
| 7 | 9.2074 | 727.1723 | 565/317 | Delphinidin-3 s malonylglucoside -5-glucoside | 1 |
| 8 | 9.9247 | 1005.2509 | 757/535/287 | Cyanidin 3-coumaroyldiglucoside-5-malonylglucoside | 1,3 |
| 9 | 10.3982 | 1005.2506 | 757/535/287 | Cyanidin 3-coumaroyldiglucoside-5-malonylglucoside | 1,3,5 |
| 10 | 10.478 | 1035.2608 | 787/535/287 | Cyanidin 3-feruloylmalonylsophoroside-5-glucoside | 1,3,5 |
| Abounding in yellow petals | | | | |  |
| ID | Ret. Time | [M + M]^+^ m/z | MS/MS (m/z) | anthocyanin |  |
| 1 | 7.091 | 789.2091 | 627/465/303 | Delphinidin-3 caffeoylglucoside -5-glucoside | 1 |
| 2 | 7.6087 | 773.2135 | 611/449/287 | Cyanidin 3-diglucoside-5-glucoside | 1,2,4 |

Note: * New anthocyanin identified in *Brassica*, which also not found in other species.

1. He, Q., Zhang, Z. and Zhang, L. (2016) Anthocyanin Accumulation, Antioxidant Ability and Stability, and a Transcriptional Analysis of Anthocyanin Biosynthesis in Purple Heading Chinese Cabbage (Brassica rapa L. ssp. pekinensis). *J Agric Food Chem* **64**, 132-145.
2. Lin, L.Z., Sun, J., Chen, P. and Harnly, J. (2011) UHPLC-PDA-ESI/HRMS/MS(n) analysis of anthocyanins, flavonol glycosides, and hydroxycinnamic acid derivatives in red mustard greens (Brassica juncea Coss variety). *J Agric Food Chem* **59**, 12059-12072.
3. Sun, J., Xiao, Z., Lin, L.Z., Lester, G.E., Wang, Q., Harnly, J.M. and Chen, P. (2013) Profiling polyphenols in five Brassica species microgreens by UHPLC-PDA-ESI/HRMS(n.). *J Agric Food Chem* **61**, 10960-10970.
4. Wu, X. and Prior, R.L. (2005) Identification and characterization of anthocyanins by high-performance liquid chromatography-electrospray ionization-tandem mass spectrometry in common foods in the United States: vegetables, nuts, and grains. *J Agric Food Chem* **53**, 3101-3113.
5. Zhang, Y., Chen, G., Dong, T., Pan, Y., Zhao, Z., Tian, S. and Hu, Z. (2014) Anthocyanin accumulation and transcriptional regulation of anthocyanin biosynthesis in purple bok choy (Brassica rapa var. chinensis). *J Agric Food Chem* **62**, 12366-12376.

**Table S6** qRT-PCR primers

| **Amplified genes** | **Sequences** |
| --- | --- |
| *TTG1* | AGTGTGAACGCGATTGCTTG (F) CAGGCAACGAAGAAGACCAC (R) |
| *GL3* | GGAGAGAAGGAGTTTTGCTTG (F) TTGATCCCTTGTGCTTGCA (R) |
| *TT8* | ATCTAATGGAGGAAGGCGGA (F) CCACTCGCTGATATTGCTGA (R) |
| *PAP2* | GCTTCTGGGAAACAGGTGGT (F) TGAAGGATCGAGGTCGAGGT (R) |
| *MYB12* | TCTGATCCAGTGGTGTCGTG (F) CAAGACAGAAGCCAAGCGAC(R) |
| *F3H* | TGACGGAGGAGTACAGCGAG (F) CATCGTCTCGTGTGGCTTGT (R) |
| *F3’H* | CCACACATCGCATCAGAGAG (F) CTCCGAACGGTATAAGCTCG (R) |
| *DFR* | TGCCGCCTAGCCTTATTACC (F) TGGCAGCAGCTTGTTCGTAC (R) |
| *ANS* | TTACATTGAAGCAACGAGTGAG (F) GCATCACAATCGAATCAGGAAC (R) |
| *TT19* | AATACTCCAACCAAGGCACG (F) ACATTCTTCGCCTAACCTGG (R) |
| *Bnaactin3* | TCCATCCATCGTCCACAG (F) GCATCATCACAAGCATCCTT (R) |
